# Isoquercitrin Alleviates Diabetic Nephropathy by Inhibiting STAT3 Phosphorylation and Dimerization

**DOI:** 10.1002/advs.202414587

**Published:** 2025-04-04

**Authors:** Chen Xuan, Donghui Chen, Shuangna Zhang, Chaofan Li, Qingyun Fang, Dinghua Chen, Jiabao Liu, Xin Jiang, Yingjie Zhang, Wanjun Shen, Guangyan Cai, Xiangmei Chen, Ping Li

**Affiliations:** ^1^ School of Basic Medical Sciences Chengdu University of Traditional Chinese Medicine Chengdu 610075 China; ^2^ Department of Nephrology First Medical Center of Chinese PLA General Hospital State Key Laboratory of Kidney Diseases National Clinical Research Center for Kidney Diseases Beijing Key Laboratory of Medical Devices and Integrated Traditional Chinese and Western Drug Development for Severe Kidney Diseases Beijing Key Laboratory of Digital Intelligent TCM for the Prevention and Treatment of Pan‐vascular Diseases Key Disciplines of National Administration of Traditional Chinese Medicine (zyyzdxk‐2023310) Beijing 100000 China; ^3^ School of Clinical Medicine Guangdong Pharmaceutical University Guangzhou 510006 China

**Keywords:** diabetic nephropathy, isoquercitrin, Iso@PEG‐GK, STAT3, SH2 domain, phosphorylation, dimerization

## Abstract

At the convergence point of multiple cytokine signals, signal transducer and activator of transcription 3 (STAT3) is a highly promising therapeutic target for diabetic nephropathy. Isoquercitrin, a natural small‐molecule inhibitor of STAT3, may have beneficial effects on diabetic nephropathy; however, the underlying mechanism remains unclear. Isoquercitrin significantly mitigated renal inflammation and fibrosis by inhibiting STAT3 activity in mice with diabetic nephropathy. Moreover, STAT3 is a direct molecular target of isoquercitrin, which as corroborated by tight and stable noncovalent binding between them. This interaction is mechanistically supported by the affinity of isoquercitrin for the Ser668–Gln635–Gln633 region within the pY+1 binding pocket of the SH2 domain. This binding obstructs pivotal processes like STAT3 phosphorylation and dimerization, thereby suppressing its transcriptional function. Finally, a kidney‐targeted nanocarrier, Iso@PEG‐GK, is developed to load isoquercitrin, thus enhancing its therapeutic precision for diabetic nephropathy. Iso@PEG‐GK significantly improved the absorption and renal distribution of isoquercitrin. This study is the first to demonstrate that isoquercitrin exerts a significant protective effect against diabetic nephropathy and may provide a novel therapeutic drug for this disease.

## Graphical abstract

This study identified a natural compound, isoquercitrin, which significantly alleviated kidney inflammation and fibrosis by inhibiting STAT3 activity. Isoquercitrin forms a strong, noncovalent bond that directly binds to STAT3. Isoquercitrin binds to the pY+1 pocket of the SH2 domain of STAT3 via hydrogen bonding with Ser668, Gln635, and Gln633, thereby blocking STAT3 phosphorylation, dimerization, and transcriptional activity.

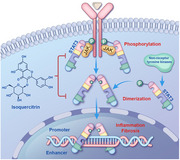

## 1 Introduction

Diabetic nephropathy, one of the most common and serious chronic microvascular complications of diabetes, is the leading cause of renal failure, with its prevalence increasing annually.^[^ ^1^ ^]^ As a chronic kidney disease now considered an epidemic, diabetic nephropathy presents significant public health challenges worldwide.^[^ ^2^ ^]^ Its pathogenesis is multifactorial, and the traditional view attributes renal injury primarily to the metabolic and hemodynamic changes caused by diabetes.^[^ ^3^ ^]^ However, this perspective has undergone a major shift, as it fails to explain why more than half of patients with diabetes do not develop diabetic nephropathy. Increasing evidence suggests that persistent inflammation and progressive fibrosis are direct contributors to diabetic kidney injury.^[^ ^4^, ^5^ ^]^

The JAK‐STAT signaling pathway, a concise mechanism, efficiently transmits signals from various cytokines, playing a pivotal role in regulating cell growth, differentiation, survival, and immunoinflammation.^[^ ^6^ ^]^ Comprising four Janus kinases (JAKs) and seven signal transducers and activators of transcription (STATs), this pathway forms the backbone of numerous cellular signaling cascades.^[^ ^7^ ^]^ Among these, aberrant activation of signal transducer and activator of transcription 3 (STAT3) is a pivotal node in the signaling cascades of multiple cytokines and a major contributor to diabetes‐induced kidney damage.^[^ ^8^, ^9^ ^]^ Aberrantly expressed cytokines, high glucose levels, and advanced glycation end products can directly or indirectly activate STAT3.^[^ ^8^ ^]^ Activated STAT3 drives the transcription of pro‐inflammatory and profibrotic cytokines, leading to intrinsic renal cell damage and extracellular matrix (ECM) accumulation.^[^ ^8^, ^9^ ^]^ In contrast, reducing STAT3 activity via genetic editing attenuates albuminuria, reduces renal macrophage infiltration, and decreases aberrant ECM accumulation in streptozotocin (STZ)‐induced diabetic nephropathy mouse models.^[^ ^9^ ^]^ Consequently, inhibiting STAT3 activity has emerged as an effective therapeutic strategy for diabetic nephropathy.^[^ ^8^, ^9^, ^10^, ^11^ ^]^

Natural products are an invaluable resource for drug discovery.^[^ ^12^ ^]^ Isoquercitrin, a natural flavonoid exhibits strong anti‐inflammatory, antioxidant, and antitumor properties.^[^ ^13^ ^]^ As a 3‐O‐glycoside derivative of quercetin, isoquercitrin has received relatively limited research attention compared to quercetin. This discrepancy may stem from its low natural concentration in plants and the complexity of its extraction process.^[^ ^14^ ^]^ However, the efficient synthesis of isoquercitrin from rutin has attracted considerable attention.^[^ ^15^, ^16^ ^]^ Isoquercitrin may have beneficial effects on diabetic nephropathy; however, the underlying mechanism remains unknown.^[^ ^17^, ^18^ ^]^ STAT3 was previously considered an undruggable target because of its smooth protein surface.^[^ ^19^ ^]^ However, the SH2 domain of STAT3 contains three ideal small‐molecule binding pockets (pY, pY‐X, and pY+1), which provide prospects for the development of small‐molecule drugs targeting STAT3.^[^ ^20^ ^]^ Furthermore, STAT3 is comprised of six structural domains, which perform different functions, and the SH2 domain mediates STAT3 phosphorylation and dimerization. STAT3 dimer formation is an important biological process after phosphorylation and is mediated by the SH2 domain.^[^ ^20^ ^]^ It remains uncertain whether isoquercitrin binds to STAT3 and what its binding site might be.

This study demonstrated that isoquercitrin exerted renoprotective effects by inhibiting STAT3 activation in endothelial and renal tubular epithelial cells. This inhibition attenuated renal inflammation and ECM accumulation in mice with diabetic nephropathy. Mechanistically, isoquercitrin bound noncovalently to the Ser668–Gln635–Gln633 sites of STAT3, occupying the pY+1 binding pocket within the SH2 domain. This interaction impedes STAT3 phosphorylation and dimerization, thereby inhibiting its transcriptional activity. Finally, to improve the absorption and renal targeting of isoquercitrin, a nanocarrier system for loading isoquercitrin was constructed. The nanocarrier optimized the absorption and renal distribution of isoquercitrin. Overall, this study demonstrated that isoquercitrin is a novel drug candidate for treating diabetic nephropathy.

## 2 Results

### 2.1 Isoquercitrin Alleviates Renal Pathological Damage in Diabetic Nephropathy and Protects Renal Function

**Figure 1:**
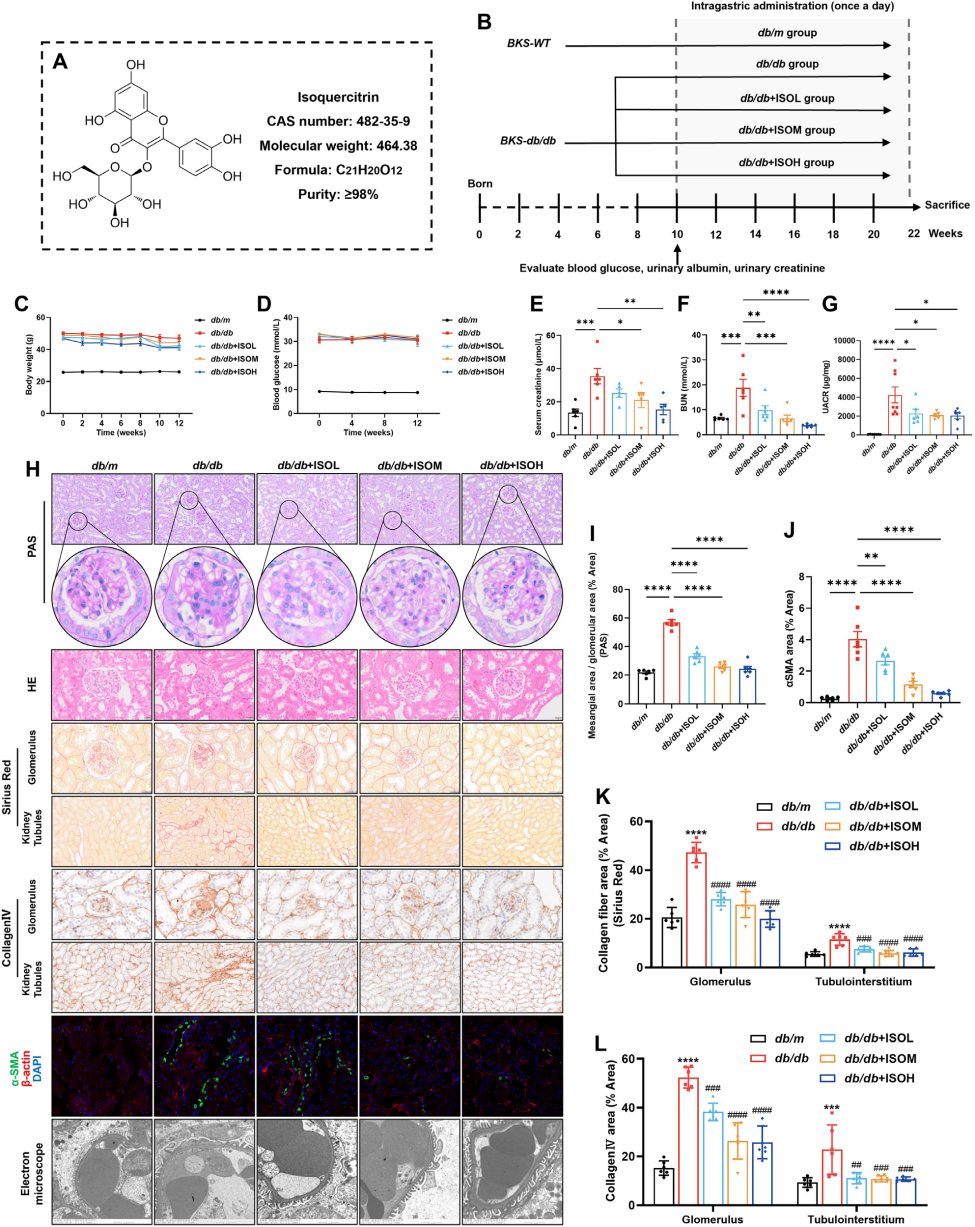
Isoquercitrin alleviates renal pathological damage and protects renal function in *db/db* mice. A) Molecular structure and information of isoquercitrin. B) Schematic overview of the experimental design of the mice model. The body weight (C) and blood glucose levels (D) of the mice were recorded during treatment with isoquercitrin (*n* = 6). Blood and urine samples were collected to detect serum creatinine (E), blood urea nitrogen (BUN) (F), and the urinary albumin/creatinine ratio (UACR) (G) at the end of the 12 weeks of isoquercitrin treatment (*n* = 6). Kidney tissues were prepared into paraffin sections and subjected to periodic acid–Schiff (PAS), hematoxylin and eosin (HE), and Sirius Red staining (H). The mesangial area (I) and collagen fiber area (K) were calculated. Immunohistochemistry and immunofluorescence detection of the expression of collagen IV (H, L) and alpha‐smooth muscle actin (α‐SMA) (H, J) in kidney tissues (*n* = 6). H) Electron microscopy observation of pathological changes of podocytes and the basement membrane (*n* = 3). Data are presented as the mean ± standard error of the mean (SEM). *p < 0.05, **/^##^ *p* < 0.01, ***/^###^ *p* < 0.001, ****/^####^ *p* < 0.0001. In (K) and (L), “*” indicates comparison with *db/m* mice, and “^#^” indicates comparison with *db/db* mice. One‐way ANOVA followed by the Dunnett's post hoc test.

The molecular structure of isoquercitrin is shown in **Figure** **1**A. A schematic overview of the experimental design of the mouse model is shown in Figure 1B. All *BKS‐db/db* (*db/db*) mice had a stable body weight and blood glucose level of >20 mmol L^−1^ at 10 weeks of age and remained stable for the next 12 weeks. Notably, isoquercitrin did not affect blood glucose or body weight in *db/db* mice (Figure 1C,D). This finding suggests that isoquercitrin may act directly on the renal tissue. Serum creatinine and blood urea nitrogen (BUN) levels were significantly higher in *db/db* mice than in *BKS‐WT* (*db/m*) mice. Isoquercitrin reduced serum creatinine and BUN levels and improved renal function dose‐dependently (Figure 1E,F). The concentration ratio of albumin‐to‐creatinine in the urine of *db/db* mice was much higher than that in the urine of *db/m* mice, suggesting increased renal albumin excretion. Isoquercitrin treatment significantly decreased the urinary albumin/creatinine ratio (UACR) (Figure 1G).

Periodic acid–Schiff (PAS), hematoxylin and eosin (HE), and Sirius red staining were used to evaluate renal pathological changes. Significant glomerular mesangial cell proliferation, mesangial matrix expansion, basement membrane thickening, loss of the brush border of tubular epithelial cells, and tubulointerstitial ECM accumulation were observed in the kidneys of *db/db* mice. Isoquercitrin significantly ameliorated these pathological injuries (Figure 1H,I,K). Collagen IV, a major component of the ECM, accumulates extensively in the glomeruli and the peritubular interstitium in the kidneys of *db/db* mice, thereby disrupting the structures of both regions. This was notably improved in the isoquercitrin‐treated groups (Figure 1H,L). The fibroblast marker alpha‐smooth muscle actin (α‐SMA) was highly expressed in the tubular epithelial cells of *db/db* mice, suggesting an abnormal transition from epithelial to mesenchymal cells. Isoquercitrin inhibited this aberrant transformation dose‐dependently (Figure 1H,J). Additionally, electron microscopy revealed podocyte foot process fusion and basement membrane thickening in *db/db* mice, whereas mice treated with isoquercitrin exhibited healthier podocyte and basement membrane morphology (Figure 1H, bottom).

A drug toxicity experiment was conducted to evaluate the potential toxicity of isoquercitrin. An animal intervention was carried out for 12 weeks using doses of 40, 80, and 120 mg kg^−1^ of isoquercitrin, and heart, liver, and spleen tissues, which are susceptible to drug toxicity damage, were collected for HE staining. The results showed that after treatment with the three different doses of isoquercitrin, there were no significant abnormal changes observed in the hearts, livers, and spleens of the mice (Figure S1, Supporting Information). This suggests that isoquercitrin has good medication safety.

### 2.2 Isoquercitrin Targets the JAK‐STAT Pathway, Regulating the Immune‐Inflammatory Response

**Figure 2:**
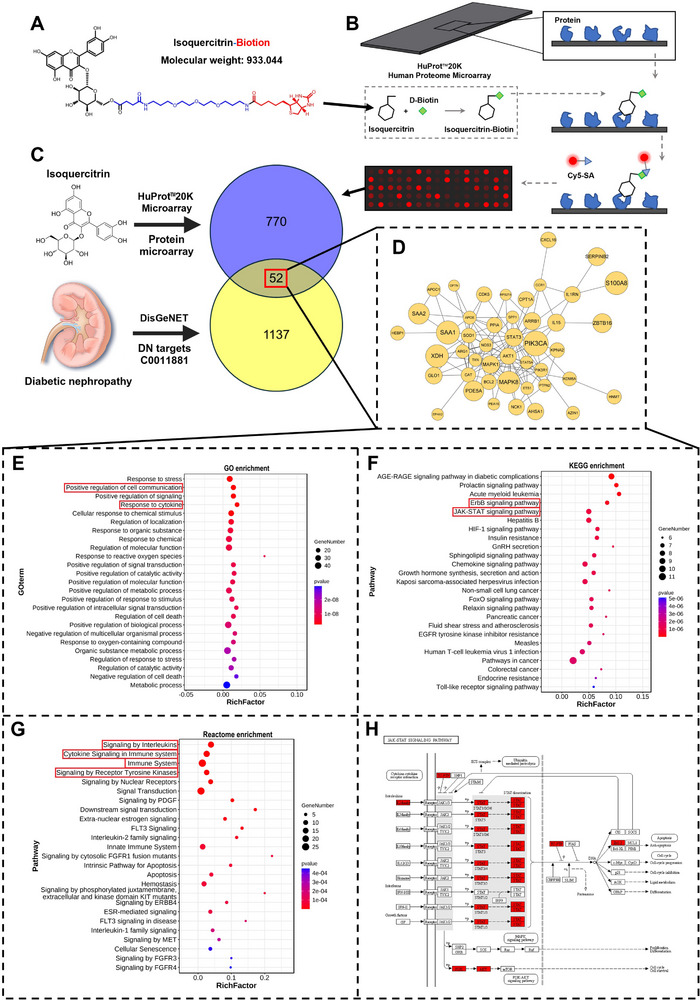
Isoquercitrin targets the JAK‐STAT pathway, thereby regulating the immune‐inflammatory response. Isoquercitrin labeled with D‐biotin (A) was incubated with the HuProt™20K human proteome microarray. B) Workflow of the proteome microarray. C) The significantly specific binding proteins of isoquercitrin screened by the proteome microarray were intersected with diabetic nephropathy disease targets. The common targets (D) were extracted and subjected to Gene Ontology (E), Kyoto Encyclopedia of Genes and Genomes (F), and Reactome (G) enrichment analysis. H) The common targets were mapped onto the JAK‐STAT signaling pathway.

The potential isoquercitrin targets were identified to explore its mechanism for treating diabetic nephropathy. D‐biotin‐labeled isoquercitrin (**Figure** **2**A) or D‐biotin was incubated with the microarray. Subsequently, streptavidin‐conjugated Cy5 was used to detect isoquercitrin‐binding proteins (Figure 2B). Among over 20 000 human proteins, 822 showed a significant Cy5 signal (Figure 2C, above) and bound to isoquercitrin to varying degrees. In the microarray results, the proteins showing the strongest binding affinity to isoquercitrin belong to a class of metallothionein proteins. However, there is no clear evidence indicating that these proteins are associated with diabetic nephropathy. By intersecting these proteins with 1189 proteins, which are related to diabetic nephropathy from the DisGeNET database (Figure 2C, below), 52 proteins were obtained (Figure 2C, middle, 2D). Isoquercitrin binds to these proteins, which are implicated in diabetic nephropathy progression and contribute to its therapeutic benefits.

Bioinformatic methods were used to analyze the biological functions of these 52 proteins. Gene Ontology‐Biological Process enrichment analysis suggested that these proteins were related to intercellular communication and cellular responses to cytokines (Figure 2E). The top five entries in the Kyoto Encyclopedia of Genes and Genomes (KEGG) enrichment analysis included two inflammatory response signaling pathways, namely, the ErbB and JAK‐STAT signaling pathways (Figure 2F). The top‐ranked entries in Reactome enrichment suggested potential therapeutic targets related to interleukin (IL)‐mediated immune responses and receptor tyrosine kinase signaling (Figure 2G). Subsequently, these proteins were mapped onto the ErbB and JAK‐STAT signaling pathways to determine their specific roles in these pathways. In the ErbB signaling pathway, isoquercitrin did not target key ErbB proteins (Figure S2, Supporting Information). However, isoquercitrin directly targeted core STAT proteins in the JAK‐STAT signaling pathway (Figure 2H). Hence, focusing on the JAK‐STAT signaling pathway is essential for diabetic nephropathy using isoquercitrin as a therapeutic agent.

### 2.3 STAT3 is a Direct Binding Target for Isoquercitrin

**Figure 3:**
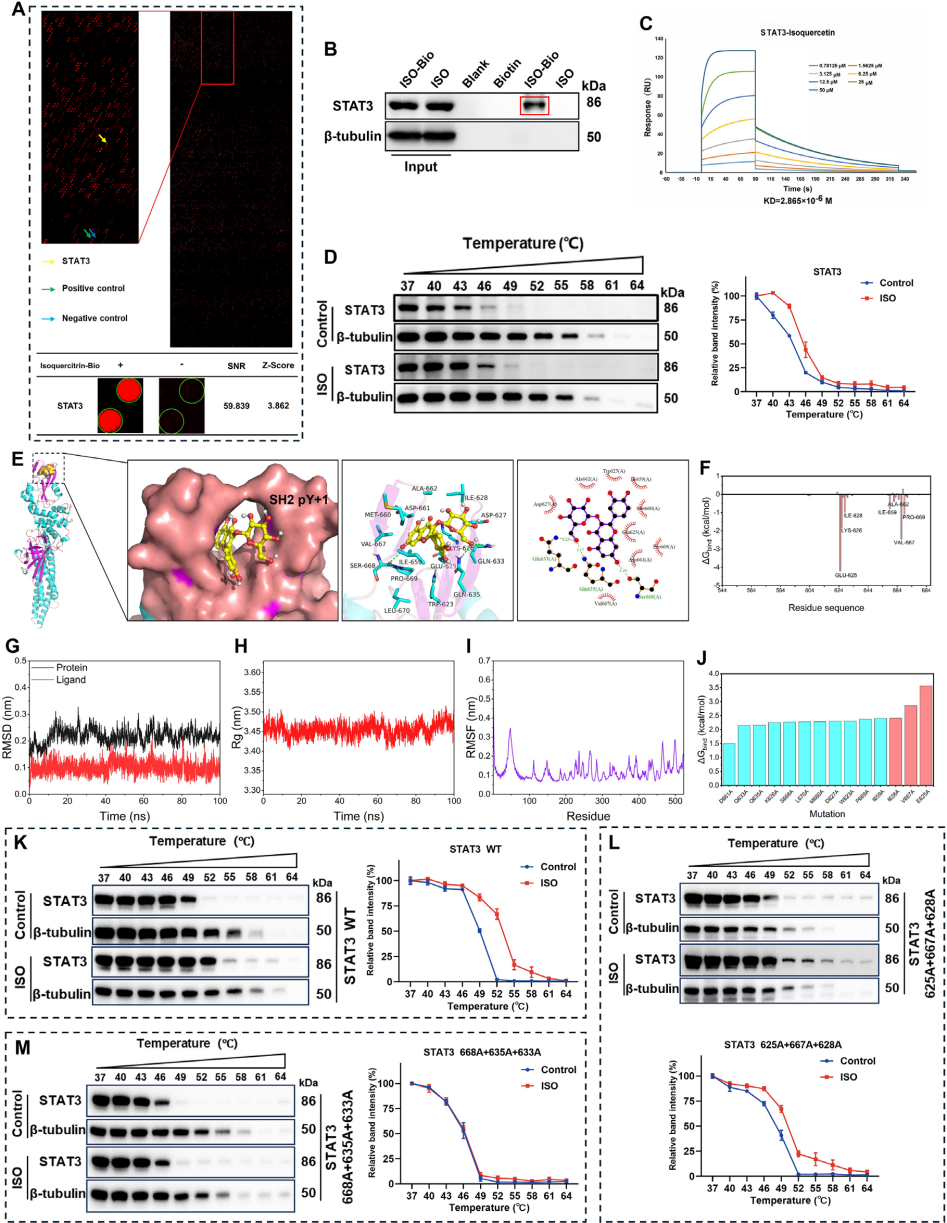
STAT3 is a direct binding target for isoquercitrin, with isoquercitrin specifically binding to the Ser668–Gln635–Gln633 site of STAT3. A) STAT3 in proteome microarray results. B) Pull‐down experiments to detect the binding of isoquercitrin to STAT3 (*n* = 3). C) Surface plasmon resonance precisely measures the binding affinity and mode of isoquercitrin to STAT3. D) Cellular thermal shift assay (CETSA) confirms whether isoquercitrin can enter cells and bind to STAT3 (*n* = 3). E) Molecular docking and dynamics simulations assessing the binding mode of isoquercitrin with STAT3. F) The MM/GBSA method calculates the contributions of different amino acid residues in the isoquercitrin–STAT3 binding. The stability of the isoquercitrin–STAT3 binding was assessed through root‐mean‐square deviation (G), radius of gyration (H), and root‐mean‐square fluctuation (I). J) Alanine scan calculates the changes in affinity between isoquercitrin and STAT3 after mutating amino acid residues to alanine. The changes in the binding of isoquercitrin to STAT3 were detected using CETSA after mutating specific amino acids to alanine (K, L, M) (*n* = 3).

Protein microarray results showed that isoquercitrin did not bind to JAKs (Figure S3, Supporting Information), whereas tight binding was observed with STAT3. The signal‐to‐noise ratio (SNR) and Z‐score of the binding between isoquercitrin and STAT3 were 59.839 and 3.862, respectively, indicating a strong binding affinity (**Figure** **3**A). Pull‐down and surface plasmon resonance (SPR) assays were performed to evaluate the binding of isoquercitrin to STAT3. Pull‐down assay results showed that STAT3 could be pulled down by biotin‐labeled isoquercitrin, suggesting that binding occurs between the two (Figure 3B). SPR results showed that isoquercitrin rapidly bound to STAT3 when different concentrations of isoquercitrin were passed through. The KD value of the binding was 2.865 × 10^−6^ M, indicating a strong binding force. After stopping the flow of isoquercitrin, it rapidly dissociated from STAT3, suggesting that its binding may occur in a noncovalent manner (Figure 3C). STAT3 is a transcription factor in the cytoplasm and nucleus. Subsequently, whether isoquercitrin binds to STAT3 intracellularly was investigated using the cellular thermal shift assay (CETSA). Compared with dimethyl sulfoxide (DMSO), isoquercitrin significantly enhanced the thermal stability of STAT3 but did not affect β‐tubulin. This finding suggests that isoquercitrin entered the cell and specifically binds to STAT3 (Figure 3D).

### 2.4 Isoquercitrin Specifically Binds to the Ser668–Gln635–Gln633 Site of STAT3

Molecular docking and dynamic simulations were performed to elucidate the binding site of isoquercitrin to STAT3. Isoquercitrin bound to the SH2 domain of STAT3 (Figure 3E). Isoquercitrin occupies the pY+1 pocket (Figure 3E, left) by forming hydrogen bonds with three amino acid residues: Ser668, Gln635, and Gln633. The amino acid residues adjacent to isoquercitrin included Asp627, Ala662, Trp623, Ile659, Met660, Glu625, Pro669, Asp661, and Val667 (Figure 3E, right).

Subsequently, molecular dynamic simulations were performed to assess the stability of the interactions between isoquercitrin and STAT3. The root‐mean‐square deviation (RMSD) values for isoquercitrin and STAT3 fluctuated within 0.15 nm (Figure 3G), and the radius of gyration (Rg) values were ≈3.45 nm (Figure 3H), indicating that the isoquercitrin–STAT3 complex remains stable and compact. Equilibrium trajectories were selected to simulate protein–ligand interactions. The root‐mean‐square fluctuation (RMSF) values of most amino acid residues in the binding pocket were within 0.2 nm, indicating that the binding pocket remained stable during the dynamic simulation process (Figure 3I). Decomposition calculations of the binding free energy in the isoquercitrin–STAT3 complex were performed using the MM/GBSA method to identify the amino acid residues playing a key role in binding. Some STAT3 residues play essential roles in binding to isoquercitrin, including Glu625, Val667, Lys626, Pro669, and Ile628, which contribute >1.0 kcal mol^−1^ in absolute value to the binding free energy (Figure 3F). Consistent with the MM/GBSA calculation results, alanine scanning mutagenesis data indicated that the binding free energy changed significantly when some amino acid residues were mutated to alanine. Glu625, Val667, and Ile628 are the three amino acid residues that cause the greatest change in binding energy after mutation, at 3.57, 2.86, and 2.41 kcal mol^−1^, respectively (Figure 3J). Based on the above computational results, two types of STAT3 proteins were constructed with the following combined mutations: Glu625–Val667–Ile628 and Ser668–Gln635–Gln633. In STAT3‐1, Glu625, Val667, and Ile628 were simultaneously mutated to alanine, whereas in STAT3‐2, Ser668, Gln635, and Gln633 were simultaneously mutated to alanine (Figure S4, Supporting Information). Furthermore, CETSA results indicated that isoquercitrin significantly enhanced the thermal stability of STAT3 as the temperature increased in 293T cells transfected with the wild‐type STAT3 plasmid, suggesting a binding interaction (Figure 3K). The plasmid was transfected into 293T cells using the Glu625–Val667–Ile628 triple mutation. Isoquercitrin still enhanced the thermal stability of STAT3; however, this enhancement was reduced compared to wild‐type STAT3 (Figure 3L). Finally, the enhancing effect of isoquercitrin on STAT3 thermal stability completely disappeared after transfecting 293T cells with the Ser668–Gln635–Gln633 triple mutant plasmid, indicating no binding interactions (Figure 3M). Therefore, the Ser668–Gln635–Gln633 site is necessary for isoquercitrin binding to STAT3. Molecular docking, SPR, and CETSA results confirmed that isoquercitrin binds to the pY+1 pocket of STAT3 by forming hydrogen bonds with the Ser668–Gln635–Gln633 site.

### 2.5 Isoquercitrin Occupies the SH2 Domain, Inhibiting STAT3 Phosphorylation, Dimerization, and Downregulating Its Transcriptional Activity

**Figure 4:**
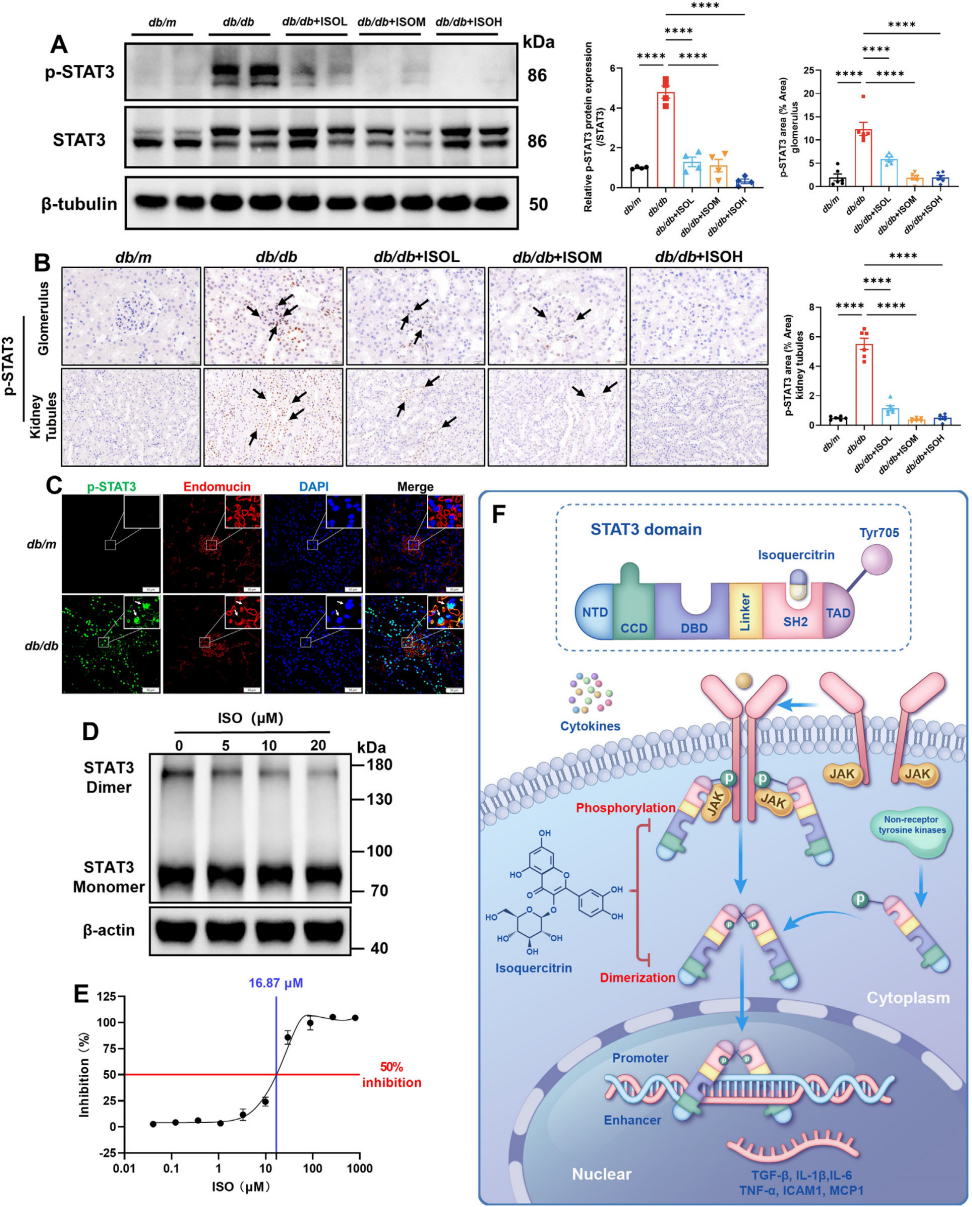
Isoquercitrin occupies the SH2 domain, inhibiting STAT3 phosphorylation, dimerization, and downregulating its transcriptional activity. A) Western blot (WB) detection of isoquercitrin effects on phosphorylated STAT3 (p‐STAT3) and total STAT3 in kidneys (*n* = 4). B) Immunohistochemistry detection of p‐STAT3 in glomerulus and kidney tubules (*n* = 6). Black arrows indicate p‐STAT3 positive regions. C) Immunofluorescence co‐staining of p‐STAT3 and the endothelial cell marker Endomucin. White arrows indicate p‐STAT3 positive regions. D) After crosslinking protein–protein interactions with DSS, the effect of isoquercitrin on STAT3 dimers in 293T cells was detected using WB. E) The determination of the half‐maximal inhibitory concentration for the inhibition of STAT3 transcriptional activity by isoquercitrin. F) Isoquercitrin inhibits STAT3 phosphorylation and dimerization. Data are presented as the mean ± SEM. ****p < 0.0001. One‐way ANOVA followed by the Dunnett's post hoc test.

Isoquercitrin occupied the pY+1 pocket in the SH2 domain by binding to the Ser668–Gln635–Gln633 site of STAT3. Further experiments were conducted to elucidate the effects of isoquercitrin on STAT3 phosphorylation and dimerization. Western blotting (WB) results suggested that phosphorylated STAT3 (p‐STAT3) expression in the kidneys of *db/db* mice was significantly increased. Isoquercitrin significantly reduced STAT3 phosphorylation levels (**Figure** **4**A). Immunohistochemical analysis confirmed that p‐STAT3 was abundantly expressed in the glomeruli and tubules of *db/db* mice, whereas only a small amount of p‐STAT3 was expressed in the kidneys of *db/m* mice. Isoquercitrin significantly inhibited p‐STAT3 expression in both the glomeruli and renal tubules (Figure 4B). Immunohistochemical analysis showed a significant increase in p‐STAT3 expression in renal tubular epithelial cells of *db/db* mice (Figure 4B, below). Owing to the damaged structure of the glomeruli in *db/db* mice, whether similar STAT3 phosphorylation also occurs in endothelial cells could not be determined (Figure 4B, above). Therefore, immunofluorescence co‐staining for p‐STAT3 with endothelial cell marker Endomucin was performed. The results showed that p‐STAT3 overlapped with Endomucin‐positive cells, suggesting that STAT3 was also phosphorylated in endothelial cells (Figure 4C). Immunohistochemistry and immunofluorescence indicated that isoquercitrin inhibited the abnormally elevated STAT3 phosphorylation levels in the renal endothelial cells of *db/db* mice. After the protein interactions were fixed using the DSS Crosslinker, the WB results showed that isoquercitrin inhibited the formation of the STAT3 dimer in a dose‐dependent manner (Figure 4D).

Isoquercitrin inhibited STAT3 phosphorylation and dimerization by targeting the SH2 domain. However, whether this effect ultimately translates to the inhibition of STAT3 transcriptional activity remains unclear. Therefore, a HEK Blue reporter assay was used to evaluate the effect of isoquercitrin on STAT3 transcriptional activity. The half‐maximal inhibitory concentration of isoquercitrin for inhibiting STAT3 transcriptional activity was 16.87 µM, indicating its efficacy in suppressing the transcriptional activity of STAT3 (Figure 4E). These results demonstrate that isoquercitrin is an effective STAT3 inhibitor that occupies the SH2 domain, inhibiting STAT3 phosphorylation and dimerization and reducing its transcriptional activity (Figure 4F).

### 2.6 Isoquercitrin Ameliorates Endothelial Cell and Tubular Epithelial Cell Injury by Inhibiting STAT3

**Figure 5:**
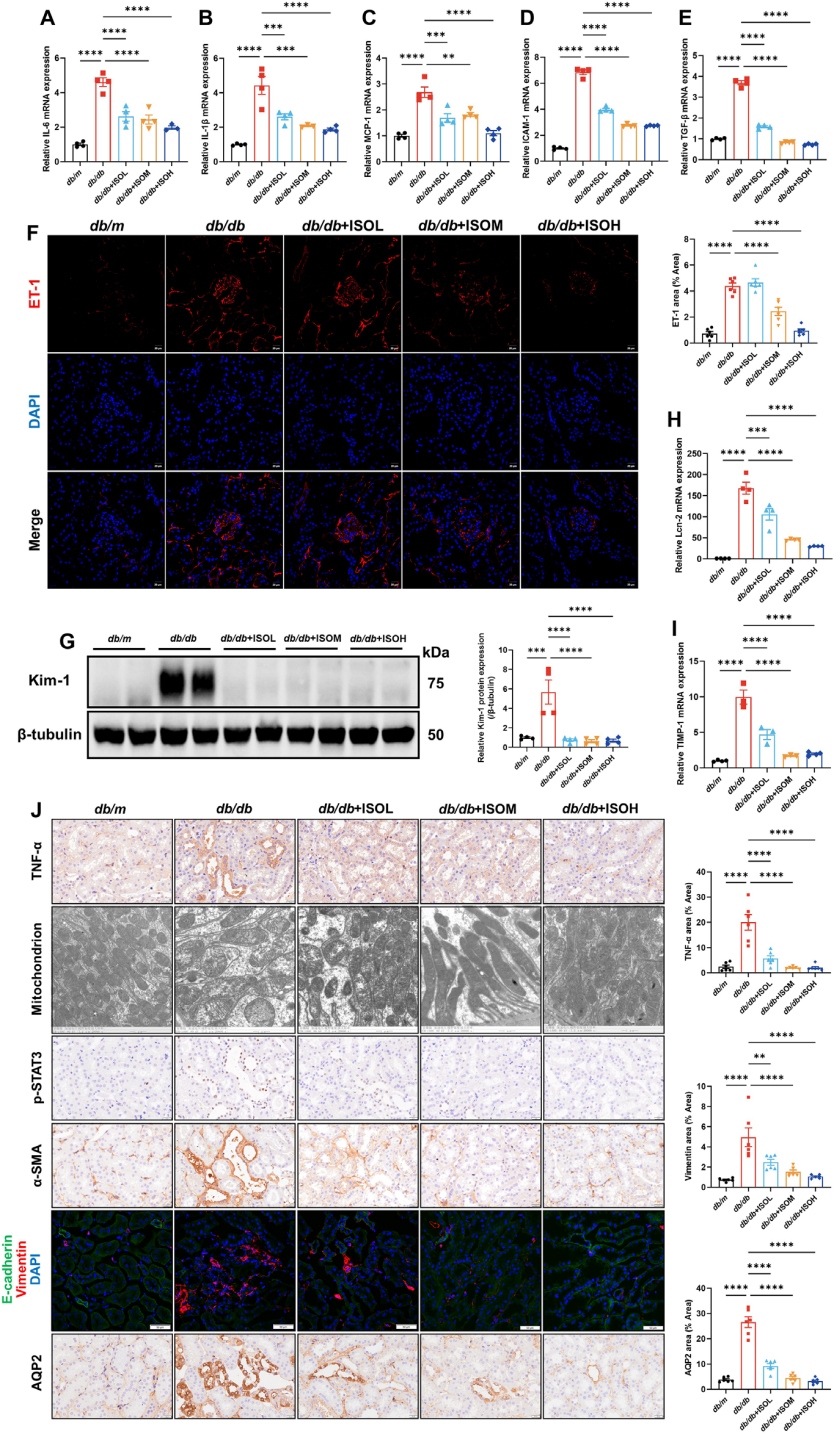
Isoquercitrin inhibits STAT3 to improve endothelial and renal tubular epithelial cell injury. A–E) RT‐qPCR detection of mRNA expression levels of interleukin (IL)‐6, IL‐1β, monocyte chemoattractant protein‐1 (MCP‐1), intercellular adhesion molecule‐1 (ICAM‐1), and tumor growth factor beta (TGF‐β) in kidneys (*n* = 4). F) Immunofluorescence detection of endothelial cell injury marker ET‐1 in kidneys (*n* = 6). G) WB detection of Kim‐1 expression, a marker of renal tubular epithelial cell injury, in mouse kidneys (*n* = 4). RT‐qPCR detection of lipocalin‐2 (Lcn‐2) (H) and tissue inhibitor of metalloproteinase 1 (TIMP‐1) (I) mRNA expression levels in kidneys (*n* = 4). J) Immunohistochemistry and immunofluorescence detection of TNF‐α, p‐STAT3, α‐SMA, E‐cadherin, vimentin, and aquaporin 2 (AQP2) expression in kidney tubules (*n* = 6). Electron microscopy observation of the morphology of mitochondria in renal tubular epithelial cells of mice (J) (*n* = 3). Data are presented as the mean ± SEM. **p < 0.01, ***p < 0.001, ****p < 0.0001. One‐way ANOVA followed by the Dunnett's post hoc test.

IL‐6 and IL‐1β are key inflammatory cytokines downstream of the STAT3 signaling pathway, and their transcription is regulated by STAT3 activation.^[^ ^21^, ^22^ ^]^ In type 2 diabetic nephropathy, IL‐6 is significantly elevated and continuously promote its own expression through a STAT3 positive feedback loop, exacerbating renal inflammation.^[^ ^23^, ^24^, ^25^ ^]^ Meanwhile, IL‐1β mediates renal inflammatory injury and disease progression by increasing endothelial cell permeability, promoting ICAM‐1 synthesis, and stimulating excessive ECM production in proximal tubular epithelial cells.^[^ ^26^, ^27^ ^]^ The transcriptional levels of IL‐6 and IL‐1β in the renal tissue of *db/db* mice were significantly increased. Isoquercitrin suppressed IL‐6 and IL‐1β mRNA expression in the kidneys of *db/db* mice (**Figure** **5**A,B). The mRNA levels of MCP‐1 and ICAM‐1 were abnormally elevated in the kidneys of *db/db* mice, and isoquercitrin treatment decreased the transcription of MCP‐1 and ICAM‐1 (Figure 5C,D). Tumor growth factor beta (TGF‐β) mRNA levels were significantly elevated in the kidneys of *db/db* mice. Isoquercitrin significantly inhibited the transcription and translation of TGF‐β (Figure 5E).

ET‐1 expression levels were significantly elevated in the kidneys of *db/db* mice. Notably, isoquercitrin reduced the aberrantly high expression of ET‐1 (Figure 5F). Kim‐1, a marker of renal tubular epithelial cell injury, was significantly elevated in the kidneys of *db/db* mice,^[^ ^28^ ^]^ and isoquercitrin significantly reduced Kim‐1 expression in the renal tissues (Figure 5G). Pro‐inflammatory cytokine tumor necrosis factor alpha (TNF‐α) is highly expressed in damaged renal tubular epithelial cells, and isoquercitrin reduced TNF‐α expression (Figure 5J). Electron microscopy indicated significant mitochondrial swelling and deformation as well as rupture and even disappearance of cristae in the renal tubular epithelial cells of *db/db* mice. However, isoquercitrin‐treated *db/db* mice showed healthier mitochondrial structures (Figure 5J). Furthermore, p‐STAT3 and α‐SMA expression in the renal tubules was observed using immunohistochemical consecutive sectioning. Damaged renal tubules highly expressed both p‐STAT3 and the fibroblast marker α‐SMA (Figure 5J). STAT3 phosphorylation in renal tubular epithelial cells may lead to their transformation into fibroblasts. Isoquercitrin, while inhibiting STAT3 phosphorylation, also reduced α‐SMA expression in renal tubular epithelial cells. In addition, E‐cadherin expression was decreased and vimentin expression was increased in the renal tubules of *db/db* mice, confirming the occurrence of epithelial–mesenchymal transition (EMT). Isoquercitrin treatment restored E‐cadherin expression and reduced vimentin overexpression (Figure 5J). Abnormal of STAT3 activation in chronic kidney disease may promote tubulointerstitial fibrosis mediated by tubular epithelial cells, potentially by upregulating the of aquaporin 2 (AQP2), lipocalin‐2 (Lcn‐2), and tissue inhibitor of metalloproteinase 1 (TIMP‐1) expression.^[^ ^29^, ^30^, ^31^ ^]^ AQP2 protein expression and Lcn‐2 and TIMP‐1 transcriptional levels were signficantly increased in the kidneys of *db/db* mice. Isoquercitrin reduced AQP2, Lcn‐2, and TIMP‐1 expression (Figure 5J,H,I).

### 2.7 Isoquercitrin Alleviates High Glucose or IL‐6‐Induced Endothelial Cell Injury by Inhibiting STAT3 Activity

**Figure 6:**
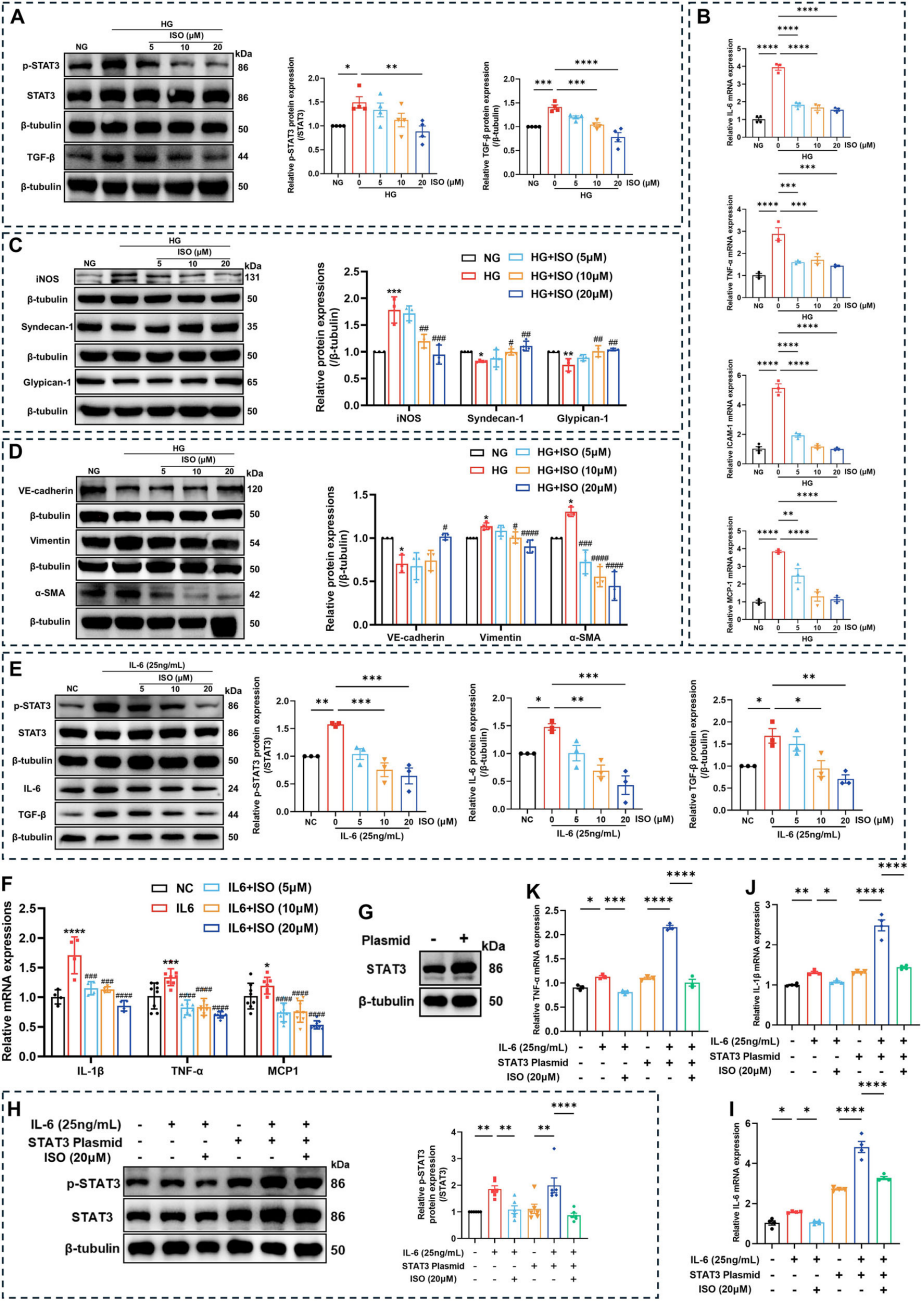
Isoquercitrin alleviates high glucose or IL‐6‐induced endothelial cell injury by inhibiting STAT3 activity. RPMI Medium 1640 containing 45 mM glucose was used as a high glucose (HG) culture medium to induce human umbilical vein endothelial cells (HUVECs). RPMI Medium 1640 with a glucose concentration of 11 mM was used to culture HUVECs in the normal glucose (NG) control group. Subsequently, isoquercitrin (ISO) at concentrations of 5, 10, and 20 µM was added to the culture medium for intervention. A) WB detection of the p‐STAT3, STAT3, and TGF‐β expression levels in HG‐stimulated HUVECs (*n* = 4). B) The mRNA expression changes of cytokines IL‐6, TNF‐α, ICAM‐1, and MCP‐1 in HUVECs after HG stimulation and ISO intervention (*n* = 3). C) WB detection of the expression of endothelial dysfunction marker inducible nitric oxide synthase (iNOS) and endothelial glycocalyx components syndecan‐1 and glypican‐1 in HUVECs (*n* = 4). D) WB detection of endothelial cell marker VE‐cadherin and mesenchymal cell markers vimentin and α‐SMA in HUVECs (*n* = 4). HUVECs were stimulated with 25 ng mL^−1^ IL‐6, whereas HUVECs without IL‐6 stimulation were the normal control (NC) group. E) WB detection of p‐STAT3, STAT3, IL‐6, and IL‐1β expression in HUVECs stimulated with IL‐6 (*n* = 3). F) The mRNA expression changes of cytokines IL‐1β, TNF‐α, and MCP‐1 in HUVECs after IL‐6 stimulation and ISO intervention (*n* = 6). STAT3 overexpression in HUVECs via plasmid transfection, with WB detection of transfection efficiency (G) (*n* = 3). H) WB detection of the effects of IL‐6 and ISO on p‐STAT3 and STAT3 expressions in HUVEC cells under transfected or nontransfected conditions (*n* = 6). I–K) RT‐qPCR detection of the effects of IL‐6 and ISO on the transcription levels of cytokines IL‐6, IL‐1β, and TNF‐α in HUVEC cells with or without transfection (*n* = 4). Data are presented as the mean ± SEM. *^/#^ *p* < 0.05, **^/##^ *p* < 0.01, ***^/###^ *p* < 0.001, ****^/####^ *p* < 0.0001. In (C), (D), and (F), * indicates comparison with *db/m*, and ^#^ indicates comparison with *db/db*. One‐way ANOVA followed by the Dunnett's post hoc test.

After growth in a high‐glucose medium, p‐STAT3 expression increased in human umbilical vein endothelial cells (HUVECs). Isoquercitrin inhibited STAT3 phosphorylation in a dose‐dependent manner in the presence of high glucose (**Figure** **6**A). In high glucose‐stimulated HUVECs, the expression of the endothelial glycocalyx components syndecan‐1 and glypican‐1 was significantly reduced, indicating charge barrier damage. Isoquercitrin restored syndecan‐1 and glypican‐1 expression, which was consistent with its effect on reducing the UACR (Figure 6C). Inducible nitric oxide synthase (iNOS) expression is increased in endothelial dysfunction induced by high glucose, and isoquercitrin similarly downregulates iNOS expression (Figure 6C).

TGF‐β expression was significantly increased in high glucose‐stimulated HUVECs (Figure 6A). High TGF‐β expression promotes endothelial cell transformation into mesenchymal cells (EndMT).^[^ ^32^ ^]^ Under high glucose conditions, the expression of the endothelial cell marker VE‐cadherin decreases, whereas vimentin and α‐SMA expression increases, indicating that EndMT occurs within the endothelial cells (Figure 6D). Isoquercitrin downregulated TGF‐β expression, thereby inhibiting EndMT in HUVECs (Figure 6A,D). In addition, the mRNA expression of pro‐inflammatory cytokines IL‐6, TNF‐α, MCP‐1, and ICAM‐1 was significantly elevated in high glucose‐stimulated HUVECs. Isoquercitrin downregulated the IL‐6, TNF‐α, MCP‐1, and ICAM‐1 mRNA expression in HUVECs (Figure 6B), consistent with its inhibition of STAT3 phosphorylation.

High glucose levels likely activate STAT3 indirectly by increasing the levels of inflammatory cytokines and signaling molecules. The transcriptional levels of IL‐6 were significantly increased in the kidneys of *db/db* mice and high glucose‐induced HUVECs (Figures 5A and 6B). Furthermore, IL‐6 was used to stimulate endothelial cells, and the effects of isoquercitrin were observed. After stimulation with 25 ng mL^−1^ IL‐6 for 4 h, the STAT3 phosphorylation level in HUVECs significantly increased. Isoquercitrin inhibited STAT3 phosphorylation in a dose‐dependent manner (Figure 6E). Concomitant with the increase in STAT3 phosphorylation levels, the IL‐6 and TGF‐β protein expression and IL‐1β, TNF‐α, and MCP‐1 transcriptional levels in HUVECs also increased. Isoquercitrin treatment downregulated the expression of these cytokines (Figure 6E,F).

HUVECs with a plasmid containing STAT3 overexpression information were transfected to elucidate the relationship between STAT3 and high expression of inflammatory cytokines. Compared to cells that did not receive plasmid transfection, STAT3 expression in the transfected HUVECs was significantly increased, indicating successful transfection (Figure 6G). Following STAT3 overexpression, the absolute p‐STAT3 content significantly increased. Furthermore, IL‐6 induced high levels of p‐STAT3 in STAT3‐overexpressing HUVECs. Notably, isoquercitrin effectively inhibited STAT3 phosphorylation induced by IL‐6 without altering the STAT3 in both transfected and nontransfected HUVEC expression. While, in STAT3‐overexpressing cells, although isoquercitrin partially inhibited STAT3 phosphorylation, the absolute p‐STAT3 content remained higher than in nontransfected cells, suggesting that the inhibitory effect of isoquercitrin may have reached saturation (Figure 6H). Compared to nontransfected cells, STAT3‐overexpressing cells exhibited higher transcriptional levels of IL‐6, IL‐1β, and TNF‐α, indicating that the transcriptional upregulation of these inflammatory factors is mediated by STAT3 (Figure 6I–K). Although isoquercitrin reduced the mRNA expression of IL‐6, IL‐1β, and TNF‐α in STAT3‐overexpressing cells, their transcriptional levels remained higher than in nontransfected cells treated with isoquercitrin (Figure 6I–K). This phenomenon is consistent with the observation that isoquercitrin‐treated transfected cells maintained high levels of absolute p‐STAT3 content compared to nontransfected cells. These results confirm that the inhibitory effect of isoquercitrin on STAT3 phosphorylation is a key mechanism in its downregulation of inflammatory cytokines.

### 2.8 Isoquercitrin Inhibits STAT3 in Renal Tubular Epithelial Cells, Reducing IL‐6‐Induced Pro‐Inflammatory and Profibrotic Cytokines

**Figure 7:**
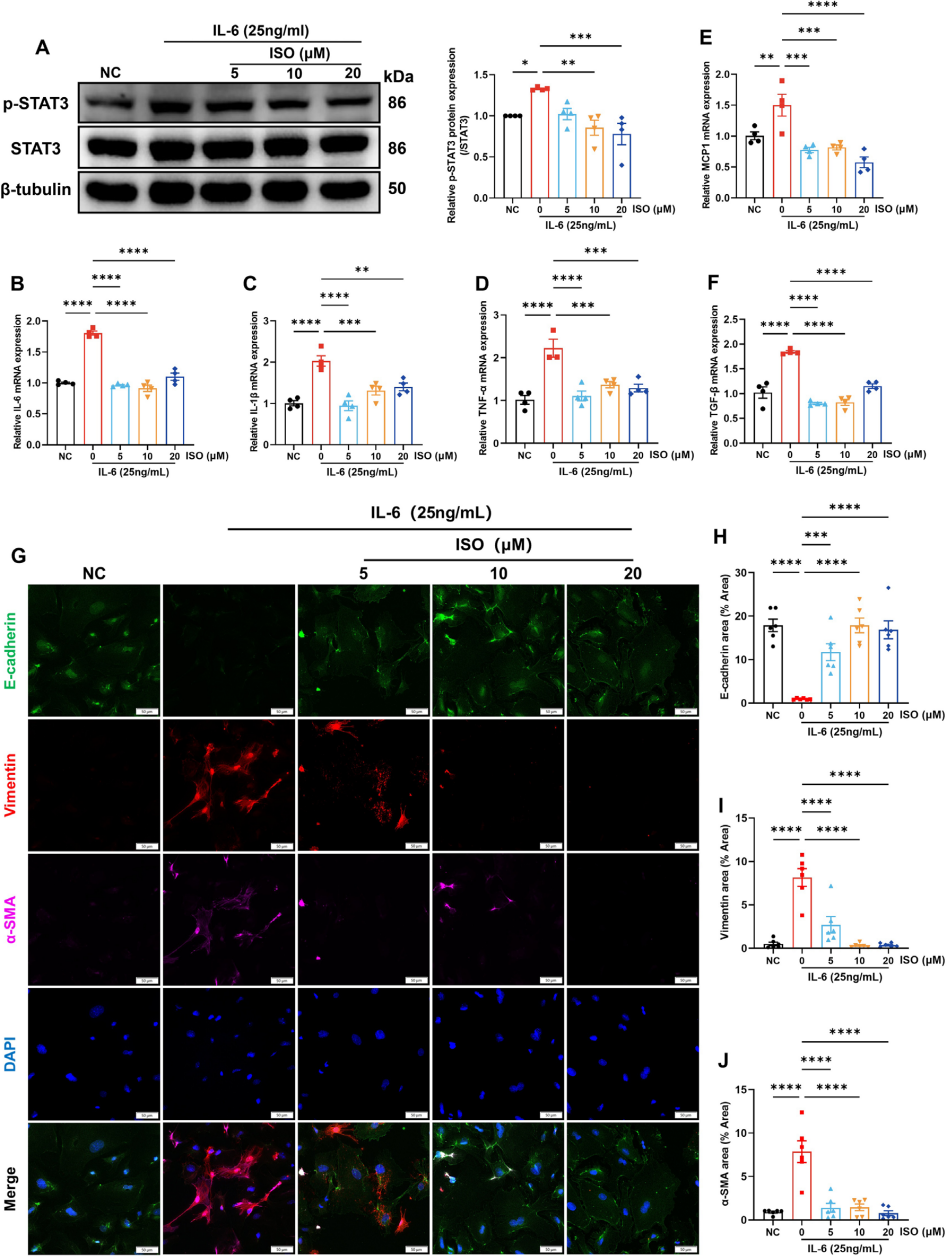
Isoquercitrin inhibits STAT3 in renal tubular epithelial cells, reducing IL‐6‐induced pro‐inflammatory and profibrotic cytokines. Human kidney 2 cells (HK2) cells were stimulated with 25 ng mL^−1^ IL‐6, whereas HK2 cells without IL‐6 stimulation were the normal control (NC) group. Subsequently, interventions were conducted with 5, 10, and 20 µM of isoquercitrin. A) WB detection of p‐STAT3 and STAT3 expression in HK2 cells (*n* = 4). RT‐qPCR detection of the mRNA expression of pro‐inflammatory cytokines IL‐6 (B), IL‐1β (C), and TNF‐α (D), macrophage chemoattractant protein MCP‐1 (E), and profibrotic cytokine TGF‐β (F) (*n* = 4). G–J) Isoquercitrin inhibits EMT in renal tubular epithelial cells. Isoquercitrin downregulates the abnormally high expression of mesenchymal markers vimentin and α‐SMA, while restoring the expression of the epithelial marker E‐cadherin (*n* = 6). Data are presented as the mean ± SEM. *p < 0.05, **p < 0.01, ***p < 0.001, ****p < 0.0001. One‐way ANOVA followed by the Dunnett's post hoc test.

Animal experiment results indicated a significant increase in p‐STAT3 expression in renal tubular epithelial cells (Figure 4B). Human kidney 2 cells (HK2) were cultured and stimulated with IL‐6 to explore the effects of isoquercitrin on renal tubular epithelial cells. This finding was consistent with the results observed in the *db/db* mice. The phosphorylation level of STAT3 in HK2 cells significantly increased after stimulation with 25 ng mL^−1^ IL‐6 for 8 h (**Figure** **7**A), accompanied by elevated IL‐6, IL‐1β, TNF‐α, MCP1, and TGF‐β mRNA expression levels (Figure 7B–F). Isoquercitrin inhibited IL‐6‐induced STAT3 phosphorylation and the abnormally high expression of these cytokines. Damaged tubular epithelial cells promote EMT by synthesizing TGF‐β, thereby exacerbating renal interstitial fibrosis.^[^ ^32^ ^]^ Immunofluorescence results showed that isoquercitrin effectively reduced the overexpression of mesenchymal markers vimentin and α‐SMA in renal tubular epithelial cells stimulated by IL‐6, while restoring the expression of the epithelial marker E‐cadherin (Figure 7G–J). These findings indicate that isoquercitrin can inhibit the EMT of renal tubular epithelial cells, which is consistent with its role in alleviating renal interstitial fibrosis of *db/db* mice.

### 2.9 Preparation and Characterization of Iso@PEG‐GK

**Figure 8:**
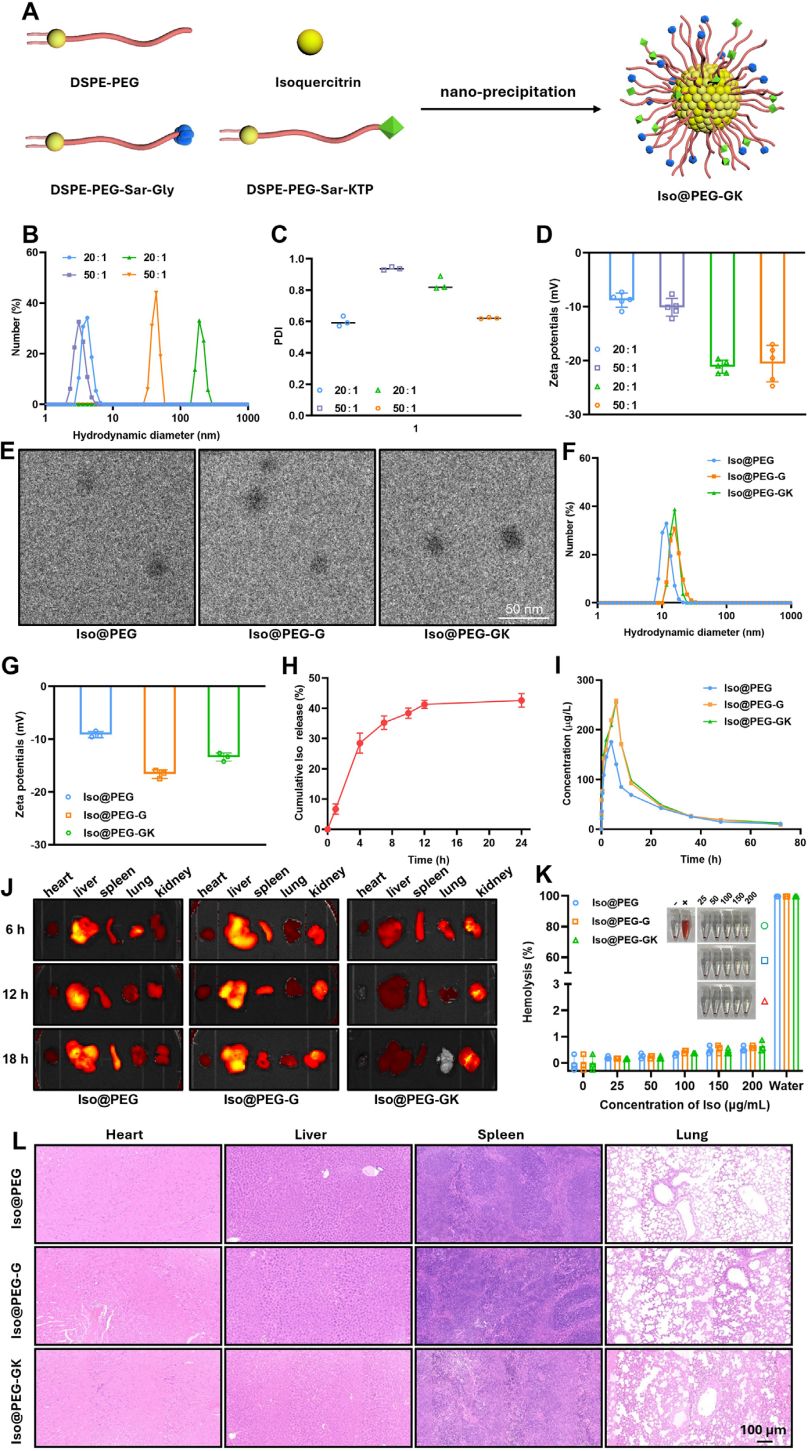
Nanocarrier Iso@PEG‐GK optimizes the pharmacokinetic properties of isoquercitrin, enhances renal targeting, and is safe. A) Synthesis process of Iso@PEG‐GK. The hydrodynamic diameter (B), polydispersity index (PDI) (C) (*n* = 3), and zeta potentials (D) (*n* = 5) of nanocarriers synthesized by the nanocoprecipitated (blue and purple) and thin film hydration (orange and green) method. E) Transmission electron microscopy observation of the shape and distribution of Iso@PEG‐GK and its control groups Iso@PEG and Iso@PEG‐G. F) Dynamic light scattering detection of the hydrodynamic size of Iso@PEG‐GK, Iso@PEG, and Iso@PEG‐G. G) The zeta potentials of nanocarriers (*n* = 3). H) The isoquercitrin release curve of Iso@PEG‐GK. I) Changes in blood drug concentration of nanocarriers within 72 h (*n* = 3). J) The distribution of Iso@PEG‐GK, Iso@PEG, and Iso@PEG‐G in the heart, liver, spleen, lungs, and kidneys at different time points (*n* = 3). K) Hemolysis assay was used to detect the red blood cell biocompatibility of nanocarriers (*n* = 3). L) HE staining were used to detect the tissue morphology of major organs after nanocarrier intervention (*n* = 3).

Two commonly used methods were employed—nanocoprecipitation and thin‐film hydration—to determine the best synthesis method for isoquercitrin nanocarriers. The synthesis of the Iso@PEG‐GK nanocarrier is shown in **Figure** **8**A. The effects of different ratios (20:1 and 40:1) of DSPE‐PEG and isoquercitrin on the performance of isoquercitrin nanoparticles were also explored. Figures 8B‐D show that the particle size of the nanocoprecipitated is much smaller than that of the thin film hydration method and has a negative potential (–10 mV) closer to the cell membrane. Therefore, the nanocoprecipitation method is more suitable than thin‐film hydration for the nanocertification of isoquercitrin. In the nanocoprecipitation method, a PEG to Iso ratio of 20:1 resulted in a smaller polydispersity index (Figure 8C). Therefore, a nanocoprecipitation method of DSPE‐PEG:Iso (20:1) was selected to synthesize Iso@PEG‐GK nanoparticles with intestinal epithelial cell‐ and kidney‐targeting functions. “G” denotes Gly‐Sar, which disguises isoquercitrin as a nutrient in the small intestine and thereby enhances its oral absorption. These characteristics enable Iso@PEG‐GK to effectively enhance the oral bioavailability of isoquercitrin.^[^ ^33^ ^]^ The “K” in Iso@PEG‐GK represents the kidney‐targeting peptide KTP. This is a highly efficient kidney‐targeting peptide with the sequence CKKEEEKKEEEKKEEEK, which has been shown to significantly enhance the kidney‐targeting ability of drugs.^[^ ^34^ ^]^ In addition, the ratio of G to K in Iso@PEG‐GK was analyzed for renal enrichment according to 0.5:1, 1:1, and 2:1, respectively. When G:K = 1:1, the enrichment effect was higher than that of the other two groups (Figure S7, Supporting Information), so G:K = 1:1 was used to explore experiments. First, transmission electron microscopy (TEM) was used to characterize the morphology of Iso@PEG‐GK and its control groups, Iso@PEG and Iso@PEG‐G. All three nanoparticles had a uniform spherical dispersion (Figure 8E), and the nanoparticle size was between 10 and 20 nm. The hydrodynamic size of the nanoparticles was measured using dynamic light scattering, and Iso@PEG (11.1 nm), Iso@PEG‐G (14.5 nm), and Iso@PEG‐GK (14.6 nm) had similar sizes (Figure 8F). Iso@PEG‐G and Iso@PEG‐GK were slightly larger than Iso@PEG because of the coating with Gly‐Sar and KTP. Gly‐Sar and KTP caused Iso@PEG‐G and Iso@PEG‐GK to have more negative potentials than Iso@PEG (Figure 8G). The release curve in Figure 8H shows that Iso@PEG‐GK slowly released 41.2% of the drug within 24 h. These results indicate the successful synthesis of Iso@PEG‐GK.

### 2.10 Pharmacokinetic Characteristics and Safety of Iso@PEG‐GK

Nanocarriers with smaller diameters exhibited higher absorption rates. Electron microscopy showed that Iso@PEG‐GK was spherical with a uniform size distribution and a diameter of <20 nm, indicating efficient absorption (Figure 8E,F). Changes in the plasma concentration of isoquercitrin were evaluated. Iso@PEG‐G and Iso@PEG‐GK had higher peak plasma concentrations than Iso@PEG. Furthermore, the half‐life of Iso@PEG was ≈8 h, whereas those of Iso@PEG‐G and Iso@PEG‐GK were extended to ≈12 h (Figure 8I). The distribution of isoquercitrin in the heart, liver, spleen, lungs, and kidneys was assessed by observing the changes in the THPP signal within organs at various time points after administration. Compared to Iso@PEG, Iso@PEG‐G and Iso@PEG‐GK showed increased renal distribution within 18 h of administration. Notably, Iso@PEG‐GK had a significantly lower distribution in the heart, liver, spleen, and lungs than Iso@PEG and Iso@PEG‐G but a higher distribution in the kidneys (Figure 8J). Through further analysis of the kidney THPP signal, we found that Iso@PEG‐GK had 20% ID g^−1^, while the targeting rate of traditional oral drugs was only 0.5–2% ID g^−1^.^[^ ^35^ ^]^ This finding suggests that Iso@PEG‐GK has better renal selectivity. Within the range of 200 µg mL^−1^, the red blood cell hemolysis rate of nanocarriers remained below 1.0%, exhibiting good biocompatibility and meeting the requirements for biomaterials used in medicine (Figure 8K). No significant morphological abnormalities were observed in the various tissues after intervention with the nanocarriers (Figure 8L).

## 3 Discussion

Diabetic nephropathy affects a large patient population but lacks satisfactory treatment options, highlighting the urgency and clinical importance of identifying effective therapeutic agents.^[^ ^1^, ^2^ ^]^ Natural products offer a rich source for drug development.^[^ ^12^ ^]^ This study confirmed the therapeutic effects of isoquercitrin on diabetic nephropathy, as evidenced by reductions in serum creatinine, BUN, and UACR, alongside the alleviation of renal pathological damage in *db/db* mice. Notably, these effects are independent  of blood sugar and body weight, indicating that isoquercitrin directly targets renal mechanisms rather than exerting systemic effects.

Evidence increasingly suggests that renal inflammation, rather than being merely a consequence, plays a pivotal role in the progression of diabetic nephropathy.^[^ ^4^, ^36^, ^37^ ^]^ As a result, the sustained renal inflammation has received increasing attention.^[^ ^37^ ^]^ The role of inflammation in diabetic nephropathy is multifaceted and involves the release of inflammatory cytokines, inflammatory signaling pathway activation, and renal intrinsic cell injury and dysfunction, collectively driving disease progression.^[^ ^4^, ^36^, ^37^ ^]^ As a key transcriptional regulator, STAT3 plays a role in various biological processes, including inflammatory responses, immune regulation, and cell proliferation.^[^ ^38^ ^]^ Abnormal STAT3 activation is implicated in kidney disease progression and mediates inflammatory cytokine signaling in various renal pathologies.^[^ ^9^, ^39^ ^]^ In the transition from acute kidney injury to chronic kidney disease, STAT3 activation promotes inflammation and fibrosis, exacerbating renal damage. STAT3 inhibition can effectively reduce the infiltration of inflammatory cells and alleviate renal inflammation.^[^ ^40^, ^41^ ^]^ Albuminuria occurs in the early stages of diabetic nephropathy, persists over a considerable period of the disease process, and contributes to its progression.^[^ ^42^ ^]^ However, reduced STAT3 activity through gene editing attenuates albuminuria, renal macrophage infiltration, and aberrant ECM accumulation in mice with STZ‐induced diabetic nephropathy. Therefore, targeting STAT3 has emerged as a promising strategy in diabetic nephropathy drug discovery.^[^ ^9^ ^]^

This study is the first to confirm that STAT3 is a direct target of isoquercitrin, as demonstrated by its direct binding to STAT3. SPR and Rg results also confirmed that this binding was tight and stable. STAT3 transports between the cytoplasm and nucleus,^[^ ^38^ ^]^ and CETSA experiments confirmed that isoquercitrin could enter cells and bind to STAT3. STAT3 exerts its biological functions through phosphorylation at tyrosine residue 705, facilitated by its SH2 domain binding to receptor or nonreceptor tyrosine kinases.^[^ ^20^ ^]^ Two p‐STAT3 molecules further recognize and bind to each other's TAD domains via the SH2 domain, forming a parallel dimer and entering the nucleus. Subsequently, the STAT3 dimer recognizes and binds to specific DNA promoter or enhancer regions through its DNA‐binding domain to regulate the transcription of IL‐1β, IL‐6, MCP‐1, ICAM‐1, and TGF‐β.^[^ ^20^, ^39^ ^]^

The three small‐molecule binding pockets in the SH2 domain are promising binding sites for the development of STAT3 inhibitors.^[^ ^20^ ^]^ The results of this study suggest that isoquercitrin binds to the Ser668–Gln635–Gln633 site of STAT3 via hydrogen bonding, specifically occupying the pY+1 pocket within the SH2 domain. The noncovalent nature of this interaction minimizes the risk of off‐target effects, which are more commonly associated with covalent inhibitors. Functional assays demonstrated that isoquercitrin inhibits STAT3 phosphorylation and dimerization, which are pivotal steps for its transcriptional activity. The HEK Blue reporter assay confirmed that isoquercitrin inhibited STAT3 transcriptional activity. These results indicate that isoquercitrin is an effective STAT3 inhibitor that inhibits the transcriptional activity of STAT3 by occupying the SH2 domain and hindering phosphorylation and dimerization processes.

STAT3 is composed of 770 amino acids forming six domains, that collectively participate in the STAT3 signaling process.^[^ ^20^ ^]^ Given the importance of STAT3 phosphorylation, dimerization and DNA binding in the entire signaling pathway, a series of inhibitors directly targeting STAT3 have been developed, with the mechanism focusing on the SH2 and DBD domains. However, small‐molecule targeting the other domains have been rarely reported.^[^ ^20^ ^]^ Currently, representative small‐molecule inhibitors of STAT3 under investigation include S3I‐201, and C48. These inhibitors bind to the SH2, or DBD domains of STAT3, thereby blocking processes of STAT3 phosphorylation, dimerization, and DNA binding.^[^ ^20^ ^]^ In this study, we found that isoquercitrin specifically binds to the SH2 domain, effectively inhibiting the phosphorylation and dimerization of STAT3. S3I‐201 is a representative STAT3 inhibitor targeting the SH2 domain. The IC50 of S3I‐201 for inhibiting STAT3 is 86 µM, whereas the IC50 of isoquercitrin is 16.87 µM. This indicates that the STAT3 inhibitory effect of isoquercitrin is significantly stronger than that of S3I‐201. It is worth noting that small molecules targeting the DBD domain may enhance the STAT3 inhibitory effect of isoquercitrin. By combining isoquercitrin with small‐molecule inhibitors targeting the DBD domain, better therapeutic outcomes may be achieved. This hypothesis warrants further investigation.

IL‐6 and IL‐1β mediate inflammatory damage in the kidney.^[^ ^21^, ^22^ ^]^ IL‐6 is a primary cytokine that activates STAT3,^[^ ^6^ ^]^ and serum IL‐6 levels of patients with type 2 diabetic nephropathy are significantly elevated, contributing to disease progression.^[^ ^23^, ^24^, ^25^ ^]^ Notably, IL‐6 can further promote its expression through a positive feedback loop by activating STAT3, thereby enhancing and sustaining renal inflammation.^[^ ^39^, ^43^ ^]^ This study revealed significantly elevated IL‐6 levels in the kidneys of *db/db* mice. However, isoquercitrin downregulated the abnormally elevated transcription levels of IL‐6, consistent with its inhibitory effect on renal STAT3. This finding suggests that the anti‐inflammatory effect of isoquercitrin on diabetic nephropathy is likely mediated by its ability to inhibit renal STAT3 activity.

Diabetic nephropathy is a secondary condition where alterations in blood and urine components caused by diabetes are significant contributors to kidney damage.^[^ ^3^, ^36^ ^]^ Renal endothelial and tubular epithelial cells form the primary barriers against such changes but are vulnerable to injury under diabetic conditions.^[^ ^44^, ^45^ ^]^ In *db/db* mice, damaged endothelial cells show increased ET‐1 synthesis, which causes endothelial dysfunction and inflammatory injury.^[^ ^46^ ^]^ Various cytokines activate STAT3, and the level of p‐STAT3 significantly increased in endothelial cells both in vivo and in vitro, possibly causing diabetic endothelial cell damage. However, isoquercitrin inhibited STAT3 phosphorylation in endothelial cells. Endothelial cells are also important components of the glomerular filtration barrier and are closely associated with albuminuria.^[^ ^47^ ^]^ Endothelial cell injury, leading to basement membrane exposure, also contributes to the development of albuminuria.^[^ ^48^ ^]^ Patients with diabetes exhibit reduced endothelial cell numbers, glycocalyx damage, and increased endothelial permeability, all of which contribute to albuminuria.^[^ ^47^ ^]^ In this study, *db/db* mice exhibited endothelial cell injury and an increased UACR, which isoquercitrin significantly ameliorated. These findings were also confirmed in HUVECs. High glucose and IL‐6 levels induce endothelial cell injury, leading to STAT3 activation and the transcription of pro‐inflammatory cytokines.^[^ ^43^, ^49^ ^]^ However, isoquercitrin inhibited STAT3 phosphorylation and downregulated the expression of these cytokines, thereby alleviating endothelial cell injury.

EndMT has been implicated in renal fibrosis in diabetic nephropathy, with up to 30–50% of FSP1/α‐SMA‐positive cells co‐expressing the endothelial cell marker CD31 in diabetic mouse models.^[^ ^50^ ^]^ Consistent with this, the results of this study showed decreased expression of the endothelial marker VE‐cadherin and increased expression of fibroblast markers vimentin and α‐SMA in high glucose‐stimulated HUVECs, indicating EndMT. However, isoquercitrin significantly inhibited the phenotypic transformation of endothelial cells, correlating with its suppression of STAT3 activity and TGF‐β expression. Furthermore, endothelial cells can mediate macrophage infiltration through MCP‐1 and ICAM‐1, thereby exacerbating renal inflammation and fibrosis.^[^ ^5^, ^51^ ^]^ In this study, MCP‐1 and ICAM‐1 transcription levels were upregulated in vivo and in vitro. However, isoquercitrin downregulated the mRNA expression of these cytokines, which may be a mechanism by which it targets STAT3 to alleviate renal inflammation. Furthermore, as a downstream factor of STAT3, IL‐1β not only increases endothelial cell permeability but also further promotes the synthesis of ICAM‐1 in endothelial cells.^[^ ^26^, ^27^ ^]^ In this study, elevated IL‐1β transcription levels were also observed in IL‐6‐stimulated HUVECs. Isoquercitrin downregulated IL‐1β transcription by inhibiting STAT3. By overexpressing STAT3, we further demonstrated that STAT3 inhibition is necessary for isoquercitrin to suppress endothelial cell‐mediated inflammation and fibrosis.

Tubular injury often occurs early in diabetic nephropathy, sometimes preceding glomerular damage.^[^ ^52^, ^53^ ^]^ In this study, STAT3 phosphorylation in renal tubular epithelial cells of *db/db* mice was significantly enhanced but inhibited by isoquercitrin. Similar results were obtained in vitro. STAT3 phosphorylation was significantly increased in IL‐6‐stimulated HK2 cells; however, isoquercitrin effectively inhibited this phosphorylation. Increased TGF‐β expression in renal tubular epithelial cells possibly leads to their EMT and the activation of renal tubular interstitial fibroblasts.^[^ ^54^ ^]^ STAT3 activation enhances TGF‐β expression, promoting EMT and fibroblast activation in renal tubular cells.^[^ ^55^ ^]^ Elevated TGF‐β mRNA expression was observed in the kidneys of *db/db* mice as well as in IL‐6‐induced HK2 cells, consistent with STAT3 activation. Renal tubules in *db/db* mice with STAT3 phosphorylation also exhibited high α‐SMA expression and loss of brush border, suggesting that STAT3 activation may promote tubular epithelial cell injury and EMT. Isoquercitrin alleviates renal tubular epithelial cell injury and inhibits their transition to mesenchymal cells by suppressing STAT3 phosphorylation. This therapeutic effect of isoquercitrin was similarly observed in cultured renal tubular epithelial cells in vitro. In addition, IL‐1β stimulates hyaluronic acid synthesis and secretion through proximal renal tubular epithelial cells, leading to an increase in ECM.^[^ ^56^ ^]^ In this study, isoquercitrin significantly downregulated IL‐1β transcript levels in HK2 cells, which may be one of the mechanisms through which isoquercitrin alleviates renal inflammation and fibrosis via STAT3 inhibition.

Finally, while isoquercitrin demonstrated considerable therapeutic potential as a natural STAT3 inhibitor. As a natural small‐molecule drug, isoquercitrin still exhibits suboptimal pharmacokinetic characteristics, characterized by low oral bioavailability (1.86%) and a lack of significant renal targeting. This limits the therapeutic effects of isoquercitrin and increases the risk of potential side effects.^[^ ^13^ ^]^ To address this limitation, a kidney‐targeted nanocarrier, Iso@PEG‐GK, was developed using a nanocoprecipitation method. This nanocarrier, encapsulated with DSPE‐PEG‐Sar‐Gly and DSPE‐PEG‐Sar‐KTP, improved isoquercitrin absorption, and enhanced renal targeting. Safety assessments confirmed the compatibility of Iso@PEG‐GK with the hematological system and major organs, making it a promising innovation in isoquercitrin‐based diabetic nephropathy therapies.

## 4 Conclusion

**Figure 9:**
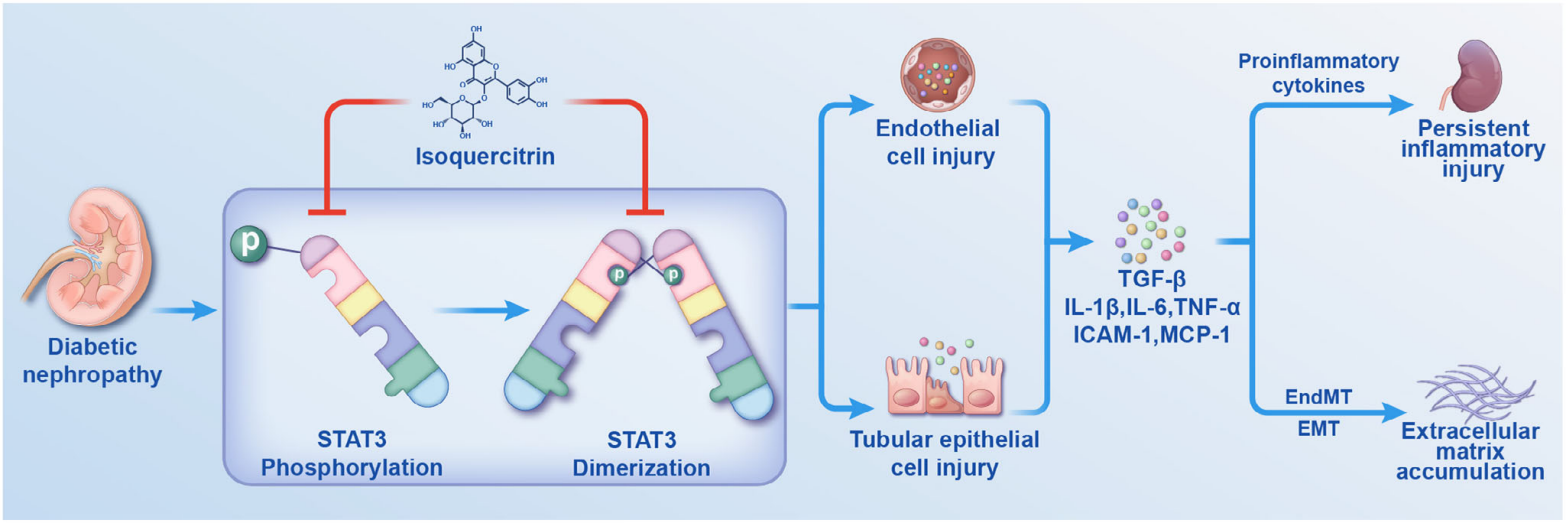
Schematic diagram showing the mechanism of isoquercitrin inhibiting STAT3 to ameliorate diabetic nephropathy. Isoquercitrin alleviates the injury of endothelial and renal tubular epithelial cells in diabetic nephropathy by inhibiting STAT3 phosphorylation and dimerization. Based on these actions, isoquercitrin reduces the expression of pro‐inflammatory and profibrotic cytokines such as IL‐1β, IL‐6, TNF‐α, ICAM‐1, MCP‐1, and TGF‐β, thereby alleviating renal inflammation and extracellular matrix accumulation.

To the best of our knowledge, this study is the first to show that isoquercitrin alleviates diabetic nephropathy by inhibiting STAT3 activity. The results of this study confirm that isoquercitrin ameliorates diabetic kidney damage by alleviating inflammation and fibrosis mediated by endothelial and renal tubular epithelial cells (**Figure** **9**). This therapeutic effect was attributed to isoquercitrin, which reduces pro‐inflammatory and profibrotic cytokines by directly binding to STAT3 and inhibiting phosphorylation and dimerization. Therefore, isoquercitrin is a promising candidate for diabetic nephropathy treatment. Furthermore, a nanocarrier was developed to optimize the pharmacokinetic properties of isoquercitrin. The results of this study provide additional information for the development of novel STAT3 inhibitors and therapeutic agents for diabetic nephropathy.

## 5 Experimental Section

### Chemicals and Reagents

Antibodies specific for Phospho‐STAT3‐Tyr705 (9145; 1:2000 for WB, 1:100 for immunofluorescence, 1:200 for immunohistochemistry), and STAT3 (4904; 1:2000 for WB) were purchased from Cell Signaling Technology (Danvers, MA, USA). Antibodies specific for vimentin (ab92547; 1:2000 for WB), collagen‐IV (ab236640;1:1000 for immunohistochemistry), α‐SMA (ab7817; 1:4000 for WB, 1:1000 for immunofluorescence, 1:20 000 immunohistochemistry) were purchased from Abcam (Cambridge, UK). Antibodies specific for syndecan‐1 (10593‐1‐AP; 1:1000 for WB), glypican‐1 (16700‐1‐AP; 1:1000 WB), BCL2 (68103‐1‐Ig; 1:5000 for WB), BAX (50599‐2‐lg; 1:2000 for WB), TGF‐β (21898‐1‐AP; 1:1000 for WB), collagen‐I (14695‐1‐AP; 1:1000 for WB), β‐tubulin (66240‐1‐Ig; 1:20 000 for WB), β‐actin (66009‐1‐Ig; 1:20 000 WB, 1:500 for immunofluorescence) were purchased from Proteintech (Wuhan, Hubei, China). Antibodies against KIM‐1 (AF1817; 1:1000 for WB) and E‐cadherin (AF748; 1:2000 for WB) were purchased from R&D Systems (Minneapolis, MN, USA). Antibodies specific for iNOS (GTX130246; 1:2000 for WB) were purchased from GeneTex (Irvine, CA, USA). The antibody specific for Endomucin (sc‐65495; 1:200 for immunofluorescence) was purchased from Santa Cruz Biotechnology, Inc (Dallas, TX, USA).

### Animal Experiments

All animal experiments were approved by the Institutional Animal Care and Use Committee (IACUC) of Cyagen Biosciences Inc. (IACUC number AACU23‐SY029). Specific pathogen‐free (SPF) *BKS‐db/db* (*db/db*) and *BKS‐wild‐type* mice (*db/m*) were housed in SPF‐grade animal facilities. The standardized housing environment included an ambient temperature of 25 ± 2 °C, 40–60% relative humidity, and a 12‐h light/dark cycle. Mice were provided with adequate water and food. Forty male *db/db* mice and eight male *db/m* mice were purchased from Cyagen Biosciences, Inc. (Suzhou, Jiangsu, China). All mice were 10 weeks old, with *db/db* and *db/m* mice weighing 42–58 and 22–25 g, respectively. At the beginning of the experiment, fasting blood glucose tests were conducted on the mice, and urine samples were collected. Diabetic nephropathy was considered established when the mice had fasting blood glucose levels of ≥16.7 mmol L^−1^, along with a significantly elevated urine albumin/creatinine ratio.^[^ ^57^ ^]^ The *db/db* mice were randomly allocated into four distinct groups: the *db/db* group and three treatment groups labeled *db/db* + ISOL (low‐dose isoquercitrin), *db/db* + ISOM (medium‐dose isoquercitrin), and *db/db* + ISOH (high‐dose isoquercitrin), with each group comprising ten mice. Eight genetically matched *db/m* mice were used as controls. In the preliminary exploration of drug dosages, it was found that treatment with 80 mg kg^−1^ of isoquercitrin showed promising therapeutic effects in mice with diabetic nephropathy (Figure S5, Supporting Information). To further investigate the dose‐dependent therapeutic effects of isoquercitrin, this study used 80 mg kg^−1^ as the medium dose for animal experiments, with 40 and 120 mg kg^−1^ designated as the low and high doses, respectively. Isoquercitrin (CAS no. 482‐35‐9, 99.13% purity; Jiangsu Yongjian Pharmaceutical Science and Technology Co., Taizhou, Jiangsu, China) was dissolved in 0.5% CMC‐Na solution and administered via gavage at low (40 mg kg^−1^), medium (80 mg kg^−1^), and high (120 mg kg^−1^) doses. Both the *db/m* and *db/db* control groups received an equivalent volume of 0.5% CMC‐Na solution via the same route. The treatments were administered once daily for 12 weeks.

### Biochemical Assays

The body weights of the mice were recorded weekly. Fasting blood glucose levels were assessed every four weeks to monitor glycemic control longitudinally. Serum creatinine (C011‐2‐1; Jiancheng Bioengineering Institute, Nanjing, Jiangsu, China) and blood urea nitrogen (BUN; C013‐2‐1; Jiancheng Bioengineering Institute) levels were quantified following the instructions provided by the assay kits. Mice were placed in metabolic cages for the collection of 24‐h urine to evaluate urinary biomarkers. Subsequently, the urine samples were centrifuged, and the resulting supernatants were analyzed for urinary albumin and creatinine levels. A creatinine (urinary) Colorimetric Assay Kit (CAY‐500701) was purchased from Cayman Chemical Company, Inc. (Ann Arbor, MI, USA). The Mouse Albumin ELISA Kit (E99‐134) for urinary albumin detection was purchased from Bethyl Laboratories, Inc. (Montgomery, TX, USA).

### Histopathological Examination

The kidneys were removed at the end of the drug intervention and rinsed in pre‐cooled saline. Half of the kidneys were fixed in a 4% paraformaldehyde solution for 48 h and embedded in paraffin. Sections (2‐µm thick) were made and evaluated pathologically using HE, PAS, and Sirius Red staining. In addition, several 1 mm^3^ renal tissues were fixed in a 2.5% glutaraldehyde solution for electron microscopic observation. OLYMPUS OlyVIA software (Hachioji‐shi, Tokyo, Japan) was used to observe and capture renal pathological changes.

### Immunohistochemistry and Immunofluorescence

Paraffin sections of kidney tissue were deparaffinized by immersion in xylene and hydrated in alcohol. Antigenic epitopes were repaired appropriately according to the manufacturer's instructions. H_2_O_2_ (3%) was used to inactivate endogenous peroxidases in tissues. After the closure of nonspecific antigenic epitopes using goat serum, sections were incubated with primary antibody at 4 °C overnight. The following day, sections were incubated with secondary antibodies at room temperature and stained with DAB. Finally, the nuclei were labeled with hematoxylin. Frozen tissue sections or cells were fixed in a 4% paraformaldehyde solution. Permeabilization was selectively performed using 0.2% Triton X‐100, depending on the site of protein expression. After sealing the nonspecific antigenic epitopes with 5% goat serum, the tissues or cells were incubated with appropriate concentrations of primary and secondary antibodies. Finally, cell nuclei were stained with 4′,6‐diamidino‐2‐phenylindole.

### Protein Microarray Assay

Protein microarrays are well suited for the rapid screening of isoquercitrin‐binding proteins because of their high‐throughput detection. The HuProt20K Human Proteome Microarray was used to search for isoquercitrin targets, which is the highest throughput human protein microarray, with a coverage of ≈20 000 full‐length human proteins.^[^ ^58^ ^]^ Recombinant proteins were GST‐tagged, expressed, and purified using eukaryotic expression systems, and each protein on the chip had a repeat site.^[^ ^58^ ^]^ At the beginning of the experiment, isoquercitrin was labeled using D‐biotin, and D‐biotin was used as a control. Isoquercitrin and D‐biotin were dissolved in DMSO and diluted in phosphate‐buffered saline to prepare a working solution. Microarray hybridization, washing, and detection were performed according to standard microarray detection procedures.

### Bioinformatics Analysis

DisGeNET is one of the largest available human disease databases. DisGeNET integrates data from public databases, genome‐wide association studies catalogs, animal models, and scientific literature to provide a reliable basis for human disease research and drug development.^[^ ^59^ ^]^ The disease targets of diabetic nephropathy (C0011881) in this study were obtained from the DisGeNET database. From the protein microarray results, isoquercitrin‐specific binding proteins were screened with a Z‐Score ≥ 2.8 and intersected with diabetic nephropathy targets to obtain possible therapeutic isoquercitrin targets. These targets were then entered into the STRING platform for GO, KEGG, and Reactome enrichment analysis.^[^ ^60^ ^]^

### Molecular Docking and Dynamics Simulation

AutoDock Vina is a widely used molecular docking program.^[^ ^61^ ^]^ The crystal structure of the STAT3 protein used for docking was obtained from the PDB database^[^ ^62^ ^]^ (6NJS), and the 3D structure of isoquercitrin was obtained from the PubChem database.^[^ ^63^ ^]^ Molecular energies were minimized under the MMFF94 force field, and molecular docking was performed using AutoDock Vina 1.2 software. The isoquercitrin‐STAT3 complex obtained based on docking was used as the initial structure for all‐atom molecular dynamics simulations, which were performed using AMBER 20 software.^[^ ^64^ ^]^ Small molecules and proteins were described using the GAFF2 small‐molecule force and ff14SB protein force fields, respectively. Subsequently, the MM/GBSA method was used to calculate the binding free energy between the protein and ligand and perform alanine scans. MD trajectories of 90–100 ns were used as calculations in this study because long molecular dynamics simulations may be detrimental to the accuracy of MM/GBSA calculations.

### Surface Plasmon Resonance

SPR analyzed the equilibrium, thermodynamics, and rate constants of intermolecular interactions and was used to accurately assess the affinity between target proteins and small‐molecule compounds.^[^ ^65^ ^]^ The BIAcore T200 instrument was used to perform SPR assays. Wild‐type STAT3 proteins were diluted to 30 µg mL^−1^ using 10 mM acetate (pH 4.5). The proteins were then immobilized on the surface of the CM5 chip at a flow rate of 10 µL min^−1^ for 600 s. Proteins were analyzed using a BIAcore T200 instrument.^[^ ^66^ ^]^ The binding of isoquercitrin to STAT3 was also investigated.

### Cell Culture and Treatment

HUVECs are ideal cell lines for modeling renal endothelial cell injury and are sensitive to high glucose stimulation.^[^ ^47^ ^]^ HUVECs, HK2, and HEK293T cells were obtained from the American Typical Culture Collection (Manassas, VA, USA). HUVECs were used in RPMI Medium 1640 (C11875500BT; Gibco, Carlsbad, CA, USA), HK2 cells in Dulbecco's modified Eagle's medium Nutrient Mixture F‐12 medium (C11330500BT; (Gibco), and HEK293T cells in Dulbecco's modified Eagle's medium (C11995500BT; Gibco). All media were supplemented with 10% fetal bovine serum (CG1126B; CELLiGENT, Hamilton, New Zealand), and cells were cultured in an incubator at 37 °C and 5% CO_2_. Cell culture grade D‐(+)‐glucose (Sigma‐Aldrich, St. Louis, MO, USA) was used to prepare the high glucose medium. Unless otherwise noted, the high glucose concentration was 45 mM, and the IL‐6 concentration was 25 ng mL^−1^ throughout the study. The results of the CCK‐8 assay indicated that when the concentration of isoquercitrin exceeded 100 µM, the viability of both endothelial cells and renal tubular epithelial cells was significantly reduced (Figure S5, Supporting Information). Further dose exploration revealed that within the range of 5, 10, and 20 µM, isoquercitrin stably inhibited the phosphorylation of STAT3 in a dose‐dependent manner. The IC50 of isoquercitrin for inhibiting STAT3 activity was 16.87 µM, which was consistent with its dose range for inhibiting STAT3 phosphorylation (Figure 4E). Therefore, in this study, the concentrations of isoquercitrin used in cell experiments were set at 5, 10, and 20 µM. All cell experiments were independently repeated thrice.

### Cell Counting Kit‐8

The Cell Counting Kit‐8 (CCK‐8; CK04) was purchased from DOJINDO Laboratories (Kumamoto, Japan). HUVECs were seeded in 96‐well plates at a density of 5 × 10^3^ cells/well. After the cells had adhered to the cell wall, 0, 6.25, 12.5, 25, 50, 100, and 200 µM isoquercitrin were added. After 24 h of incubation, CCK‐8 solution was added and incubated at 37 °C for 1 h. The absorbance of each well was then measured at 450 nm.

### Cell Transfection

Cells were seeded in a 6‐well plate and transfected with Lipofectamine 3000 reagent until they reached 70% confluence. The plasmid was diluted using 125 µL of Opti‐MEM medium according to the manufacturer‘s instructions, and 5 µL of P3000 reagent was added and mixed well. The diluted plasmid was added to the Lipofectamine 3000 reagent and incubated for 5 min. The mixture was added to the cells and incubated for 24 h. The cells were collected for subsequent experiments.

### Pull‐Down Assay

For pull‐down experiments, biotin‐labeled isoquercitrin was first co‐incubated with streptavidin agarose beads for 1 h at room temperature, while biotin was used as a control. Subsequently, 293T cell lysates overexpressing STAT3 were added and incubated overnight for 4 h. The next day, the eluent was added after three washes in TBS and eluted for 5 min in a 95 °C water bath. Protein samples were separated using sodium dodecyl sulfate‐polyacrylamide gel electrophoresis (SDS‐PAGE), and STAT3 content was detected by WB.

### Cellular Thermal Shift Assay

CETSA was performed using fixed isoquercitrin concentrations at various temperatures.^[^ ^67^ ^]^ 293T cells overexpressing STAT3 were treated with 100 µM isoquercitrin and equal volumes of DMSO for 2 h. Subsequently, cell precipitates were collected and heated on a thermal cycler (Veriti 96 Wells thermal cycler, Thermo Fisher Scientific, Waltham, MA, USA) for 3 min at 37, 40, 43, 46, 49, 52, 55, 58, 61, 64, 67, and 70 °C and immediately removed and left at room temperature for 3 min. Proteins were extracted by repeatedly freezing and thawing the cells in liquid nitrogen, and STAT3 content was detected by WB.^[^ ^68^ ^]^

### STAT3 HEK Blue Reporter Assay

The inhibitory effect of isoquercitrin on STAT3 transcriptional activity was detected using the HEK Blue Reporter system. Isoquercitrin was prepared at a maximum concentration of 800 µM and serially diluted through a 3‐fold dilution to ten concentrations using a TECAN EVO200. Different isoquercitrin concentrations were transferred to 384‐well plates using Echo655 and inoculated with 45 uL of cell suspension (12 000 cells/well) and 5 µL of IL‐6. The plates were incubated overnight in a 37 °C incubator. The next day, different buffers were added separately according to the manufacturer's instructions, and secreted alkaline phosphatase levels were measured at 620 nm using a spectrophotometer.

### STAT3 Dimer Detection

The cells were co‐incubated with different isoquercitrin concentrations (5, 10, and 20 µmol L^−1^) or 0.1% DMSO for 24 h when 293T cells were grown to a suitable density. Then, nondenaturing cell lysates were used to extract total protein. After adjusting the protein concentration to 12 mg mL^−1^, 5 mM of DSS was added and incubated at 4 °C for 1 h to immobilize protein interactions. The crosslinking reaction was terminated using Tris buffer (pH 7.5). SDS‐PAGE and WB were used to separate and assay the STAT3 dimers and monomers.

### Quantitative Teal‐Time PCR

Total RNA from kidney tissues and cells was extracted using TRIzol reagent and reverse transcribed to cDNA. SYBR Green kits and Applied Biosystems 7500 system were used for quantitative real‐time PCR assays.^[^ ^69^ ^]^ Primer sequences used in this study are listed in Table S1 (Supporting Information).

### Western Blotting

Total proteins from tissues and cells were extracted using RIPA lysis buffer, and protein concentrations were assayed using a BCA kit. Proteins were separated using SDS‐PAGE according to their molecular weights and subsequently transferred to nitrocellulose (NC) membranes. After sealing the nonspecific antigenic epitopes, the proteins attached to the NC membrane were labeled with different primary and secondary antibodies. Finally, the protein content was visualized using an ultrasensitive ECL chemiluminescent reagent and an imaging system.

### Synthetic Nanocarriers with Different Mass Ratios

Nanocarriers with different mass ratios were synthesized using film dispersion and nanoprecipitation methods. For the film‐dispersion method, 100 mg of DSPE‐PEG was weighed and dissolved in 10 mL of ethanol at 30–40 °C by mixing with a magnetic stirrer. The amount of isoquercitrin in the M1 (5 mg) and M2 (2 mg) groups were weighed and added to the polymer solution, and the drug was sonicated for 20 min to dissolve the drug. The solution was evaporated in the evaporator at 40 °C for 2 h to evaporate the organic solvent in the mixed drug–polymer solution and obtain a film. The film was hydrated by adding 10 mL of ultrapure water and sonicated for 1 h. For the nanoprecipitation method, isoquercitrin (1 mg) and DSPE‐PEG (20.0 mg) were dissolved in absolute ethanol (1 mL) to form a solution. The solution was quickly added to a mixture of water and absolute ethanol (9:1 v/v). Absolute ethanol was removed using a rotary evaporator, and the obtained product was filtered through a filter (0.22 µm) driven by a microporous syringe. The solution was washed thrice with deionized water via ultrafiltration through a centrifuge tube (Mw = 100 kDa).

### Iso@PEG‐GK Synthesis

Nanocarriers and their control counterparts were synthesized using the nanoprecipitation method. For example, isoquercitrin (1 mg), DSPE‐PEG (10.0 mg), DSPE‐PEG‐Gly‐Sar (5 mg), and DSPE‐PEG‐KTP (5 mg) were dissolved in 1 mL of absolute ethanol. The solution was quickly added to a mixture of water and absolute ethanol (9:1 v/v). Absolute ethanol was removed using a rotary evaporator, and the obtained product was filtered through a filter (0.22 µm) driven by a microporous syringe. The solution was washed thrice with deionized water via ultrafiltration through a centrifuge tube (Mw = 100 kDa). Iso@PEG (20 mg DSPE‐PEG) and Iso@PEG‐G (10 mg DSPE‐PEG‐Gly‐Sar) were used as controls.

### Characterization of Iso@PEG‐GK and Control Counterparts

Malvern Zetasizer (Nano ZS90) was used to measure the polydispersity index, hydrodynamic diameter, and zeta potential of Iso@PEG, Iso@PEG‐G, and Iso@PEG‐GK (isoquercitrin concentration = 10 µg mL^−1^). TEM (Tecnai G2) was used to characterize the nanoparticle morphology.

### Evaluation of In Vivo Targeting Accumulation and Biodistribution

Mouse models were intravenously injected with 200 µL THPP@Iso@PEG, THPP@Iso@PEG‐G, and THPP@Iso@PEG‐GK (isoquercitrin concentration = 300 µg mL^−1^). The kidney, liver, lung, spleen, and heart were used for the accumulation analysis of the isolated organs. (THPP excitation wavelength = 440 nm, emission wavelength = 670 nm, exposure time = 5 s).

### Hemolysis Evaluation and Biosafety Assessment

Erythrocytes were incubated with Iso@PEG, Iso@PEG‐G, and Iso@PEG‐GK at various concentrations for 2 h after extracting erythrocytes from mice. After centrifugation to precipitate the erythrocytes, the absorbance of the upper solution was measured at 540 nm using a multidetection microplate reader, and the hemolysis percentages of the samples were calculated. The mice were euthanized, and the liver, lung, spleen, and heart were collected for HE staining to observe the histological morphologies for safety evaluation and to evaluate the biosafety of Iso@PEG, Iso@PEG‐G, and Iso@PEG‐GK.

### Statistical Analysis

The sample size for this study was determined based on preliminary experiments, prior research, and the inherent variability of the samples. Statistical analysis was performed using GraphPad Prism software (version 9.0; GraphPad, La Jolla, CA, USA). Data are presented as the mean ± standard error of the mean. A one‐way analysis of variance was performed for multiple group comparisons. The results were considered statistically significant at *p* < 0.05.

## Conflict of Interest

The authors declare no conflict of interest.

## Author Contributions

P.L. and X.C. designed and supervised the experiments. P.L., X.C., and G.C. provided critical insights and revised the manuscript. C.X. performed most of the experiments and wrote the paper. D.C. (Donghui Chen) and S.Z. established diabetic nephropathy mice model and performed some of the experiments. C.L. and Q.F. contributed to the analysis of protein target data and performed some of the pharmacokinetics. D.C. (Dinghua Chen), J.L., and X.J. revised the manuscript. Y.Z., and W.S. performed the pathological observation. All authors read and approved the final manuscript.

## Supporting information



Supporting Information

## Data Availability

The data that support the findings of this study are available from the corresponding author upon reasonable request.
