# Supplementary material for: Isoquercitrin Alleviates Diabetic Nephropathy by Inhibiting STAT3 Phosphorylation and Dimerization
Source: Adv Sci (Weinh). 2025 Apr 4;12(25):2414587. doi: 10.1002/advs.202414587 (PMC12224983; doi:10.1002/advs.202414587)
Supplement: Supplementary file 1 — Supporting Information [file ADVS-12-2414587-s001.docx]

Supporting Information

Isoquercitrin Alleviates Diabetic Nephropathy by Inhibiting STAT3 Phosphorylation and Dimerization

Chen Xuan, Donghui Chen, Shuangna Zhang, Chaofan Li, Qingyun Fang, Dinghua Chen, Jiabao Liu, Xin Jiang, Yingjie Zhang, Wanjun Shen, Guangyan Cai*, Xiangmei Chen*, and Ping Li*

This file includes:

Table S1

Figure S1 to Figure S7

**Table S1 The RT-qPCR primer sequences used in this study.**

| **Primer name** | **Base sequence (5' to 3')** |
| --- | --- |
| human-IL1β-F | GCACCTGTACGATCACTGAACTG |
| human-IL1β-R | CCACTTGTTGCTCCATATCCTGTC |
| human-IL6-F | TTCGGTCCAGTTGCCTTCTCC |
| human-IL6-R | TCTGAAGAGGTGAGTGGCTGTC |
| human-TNFα-F | CTCATCTACTCCCAGGTCCTCTTC |
| human-TNFα-R | CGATGCGGCTGATGGTGTG |
| human-ICAM1-F | ACCTATGGCAACGACTCCTTCTC |
| human-ICAM1-R | GTGTCTCCTGGCTCTGGTTCC |
| human-MCP1-F | CCAGCAGCAAGTGTCCCAAAG |
| human-MCP1-R | TGCTTGTCCAGGTGGTCCATG |
| human-TGF-β-F | CCTGGCGATACCTCAGCAACC |
| human-TGF-β-R | CCTCCACGGCTCAACCACTG |
| human-β-actin-F | GCTTGCGGCATCCACGAGAC |
| human-β-actin-R | AGCACGGTGTTGGCATACAGATC |
| human-18S-F | CGGACAGGATTGACAGATTGATAGC |
| human-18S-R | CATGCCAGAGTCTCGTTCGTTATC |
| mouse-IL1β-F | TCGCAGCAGCACATCAACAAG |
| mouse-IL1β-R | TCCACGGGAAAGACACAGGTAG |
| mouse-IL6-F | GAGAGGAGACTTCACAGAGGATACC |
| mouse-IL6-R | TCATTTCCACGATTTCCCAGAGAAC |
| mouse-TNFα-F | CACGCTCTTCTGTCTACTGAACTTC |
| mouse-TNFα-R | CTTGGTGGTTTGTGAGTGTGAGG |
| mouse-ICAM1-F | CCACGCTACCTCTGCTCCTG |
| mouse-ICAM1-R | AAGGCTTCTCTGGGATGGATGG |
| mouse-MCP1-F | TTTGAATGTGAAGTTGACCCGTAAATC |
| mouse-MCP1-R | CTACAGAAGTGCTTGAGGTGGTTG |
| mouse-TGF-β-F | CAACAATTCCTGGCGTTACCTTGG |
| mouse-TGF-β-R | TGTATTCCGTCTCCTTGGTTCAGC |
| mouse-18s-F | CGGACACGGACAGGATTGACAG |
| mouse-18s-R | TGCCAGAGTCTCGTTCGTTATCG |
| mouse-β-actin-F | GATGGTGGGAATGGGTCAGAAGG |
| mouse-β-actin-R | TTGTAGAAGGTGTGGTGCCAGATC |
| mouse-Pdgfb-F | ATCGCACCAACGCCAACTTCC |
| mouse-Pdgfb-R | AGGCCCGGCATTGCACATTG |
| mouse-Timp1-F | AGGATTCAAGGCTGTGGGAAATGC |
| mouse-Timp1-R | CTTCACTGCGGTTCTGGGACTTG |


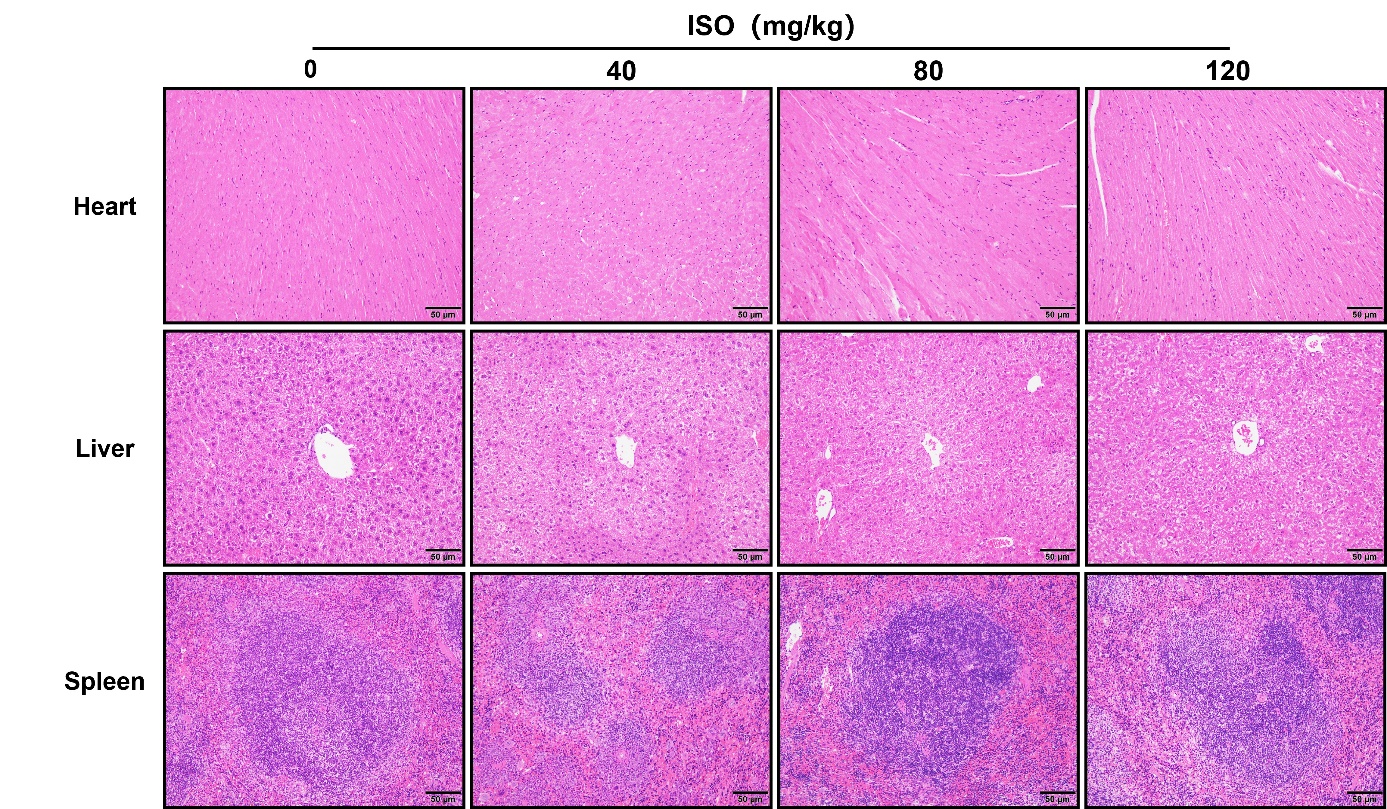


**Figure S1.** Toxicity testing of isoquercitrin. An animal intervention experiment was conducted over a 12-week period using isoquercitrin at doses of 40, 80, and 120 mg/kg. HE staining was used to observe the morphology of heart, liver, and spleen tissues (*n* = 6). Mice treated with the three different doses of isoquercitrin showed no significant abnormal damage in the morphology of heart, liver, and spleen tissues.


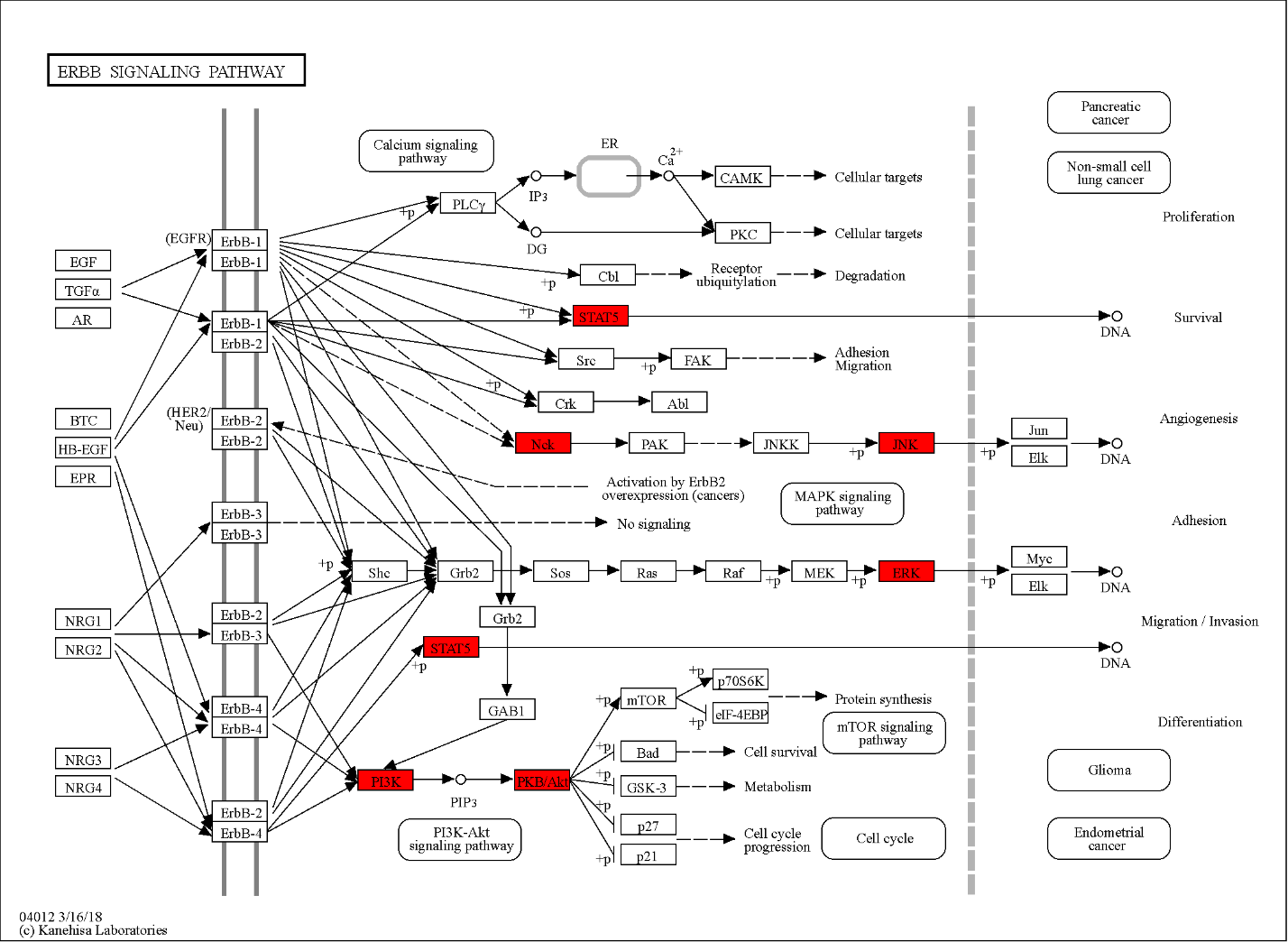


**Figure S2.** The mapping of the 52 proteins that are at the intersection of isoquercitrin specific targets and diabetes nephropathy related targets within the ErbB signaling pathway.


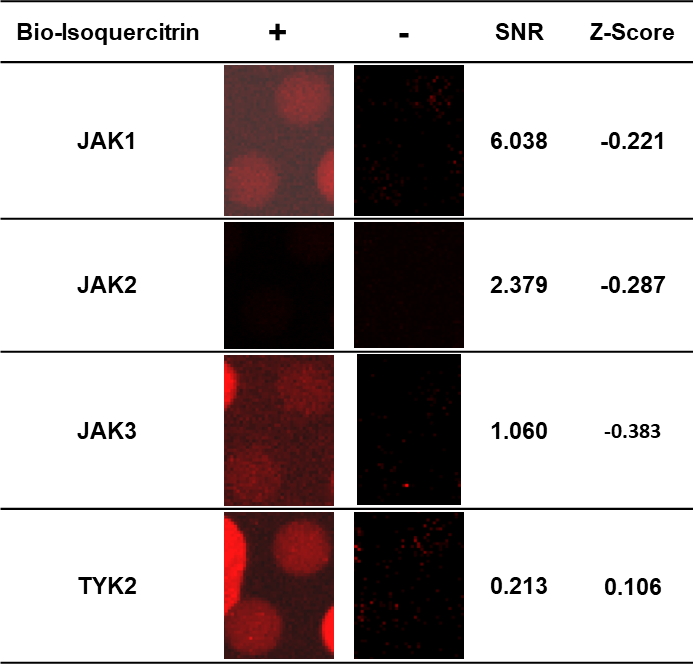


**Figure S3.** Proteome Microarray detection of the binding of isoquercitrin to JAKs. Proteome Microarray results show that isoquercitrin does not bind to any of the JAK family proteins, including JAK1, JAK2, JAK3, and TYK2.


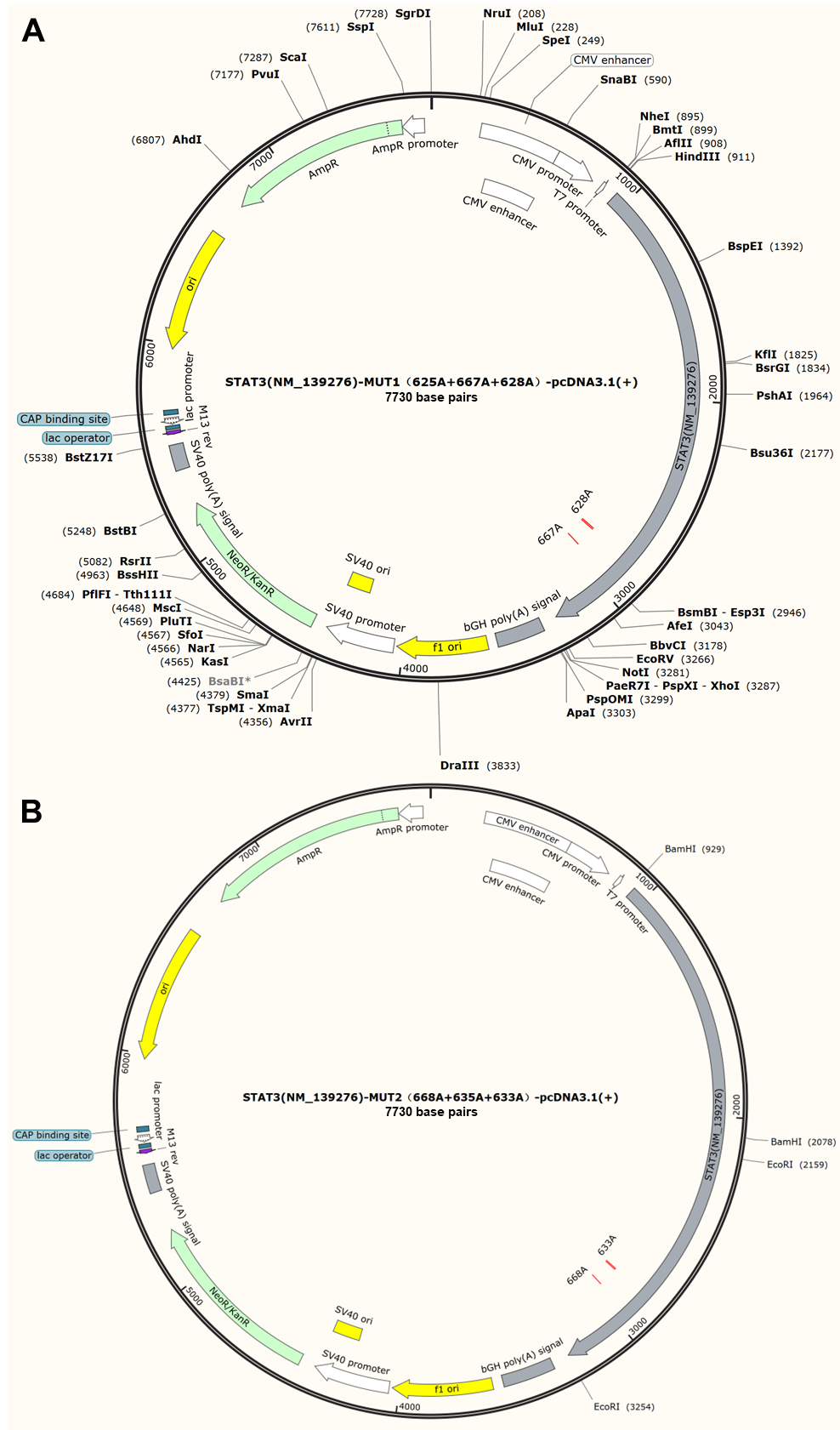


**Figure S4.** Site-directed mutagenesis of isoquercitrin's amino acids. **A** In STAT3-MUT1, Glu625, Val667, and Ile628 are mutated to alanine simultaneously. **B** In STAT3-MUT2, Ser668, Gln635, and Gln633 are mutated to alanine simultaneously.


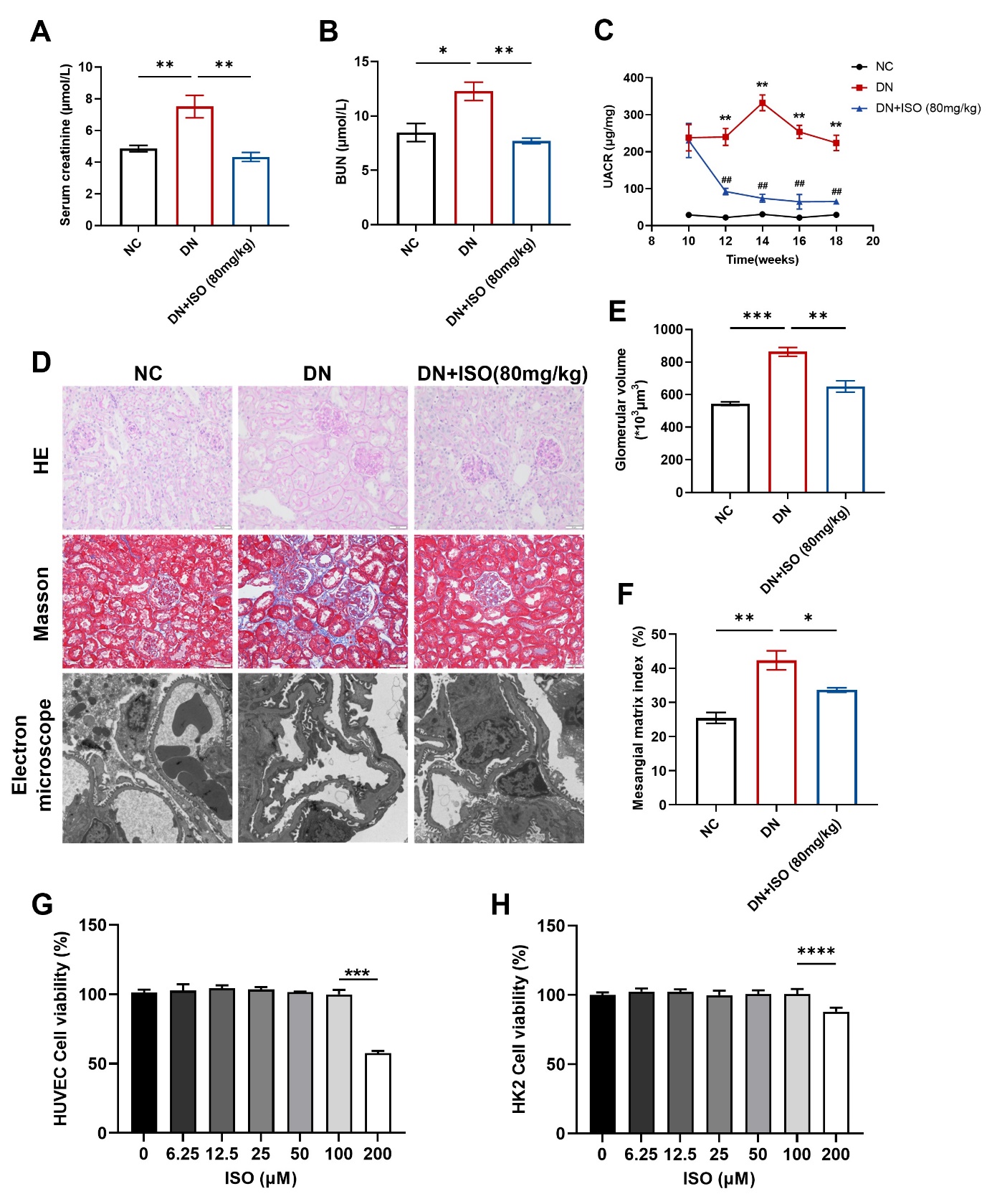


**Figure S5.** Isoquercitrin dosage exploration. **A**-**C** Serum creatinine, blood urea nitrogen (BUN), and urinary albumin/creatinine ratio (UACR) in diabetic nephropathy (DN) mice after oral administration of isoquercitrin at 80 mg/kg (*n* = 4). **D**-**F** Effects of isoquercitrin on renal pathological changes in mice (*n* = 6). **G**, **H** CCK-8 assay to evaluate the effects of isoquercitrin on the viability of HUVEC and HK2 cells (*n* = 3). Data are presented as the mean ± SEM. **p* < 0.05, ***p* < 0.01, ****p* < 0.001, *****p* < 0.0001. One-Way ANOVA followed by the Dunnett’s post hoc test.


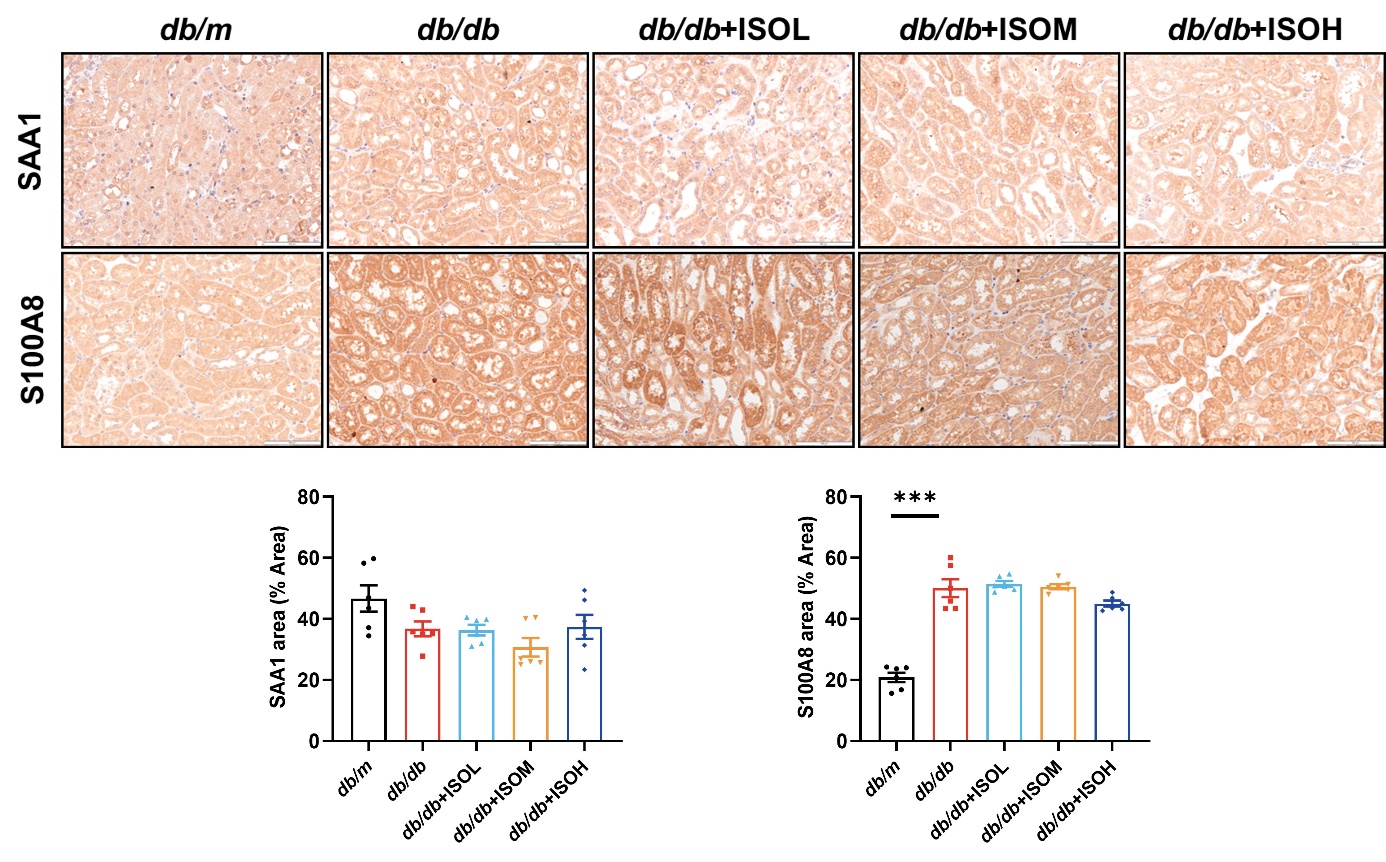


**Figure S6.** Effects of isoquercitrin on SAA1 and S100A8 in the renal tissue of *db/db* mice (*n* = 6). In addition to STAT3, isoquercitrin may also have a high binding affinity for SAA1 and S100A8. Immunohistochemical results show that isoquercitrin does not affect the expression of SAA1 and S100A8. Data are presented as the mean ± SEM. ****p* < 0.001. One-Way ANOVA followed by the Dunnett’s post hoc test.

**Figure S7.** Exploration of the ratio between DSPE-PEG-Gly-Sar and DSPE-PEG-KTP (*n* = 3). By examining three gradients 0.5:1, 1:1, and 2:1, it was found that the optimal kidney enrichment effect occurred when the ratio of DSPE-PEG-Gly-Sar to DSPE-PEG-KTP was 1:1.
